# Reinforcing the Egg-Timer: Recruitment of Novel Lophotrochozoa Homeobox Genes to Early and Late Development in the Pacific Oyster

**DOI:** 10.1093/gbe/evv018

**Published:** 2015-01-27

**Authors:** Jordi Paps, Fei Xu, Guofan Zhang, Peter W.H. Holland

**Affiliations:** ^1^Department of Zoology, University of Oxford, United Kingdom; ^2^National & Local Joint Engineering Laboratory of Ecological Mariculture, Institute of Oceanology, Chinese Academy of Sciences, Qingdao, China

**Keywords:** gene duplication, gene families, Annelida, Mollusca, Platyhelminthes, Rotifera, homeodomain

## Abstract

The metazoan superclade Lophotrochozoa includes mollusks, annelids, and several other animal phyla. It is reasonable to assume that this organismal diversity may be traced, in part, to changes in developmentally important genes, such as the homeobox genes. Although most comparative studies have focussed on ancient homeobox gene families conserved across bilaterians, there are also “novel” homeobox genes that have arisen more recently in evolution, presumably by duplication followed by radical divergence and functional change. We classify 136 homeobox genes in the genome sequence of the Pacific oyster, *Crassostrea gigas*. The genome shows an unusually low degree of homeobox gene clustering, with disruption of the NK, Hox, and ParaHox gene clusters. Among the oyster genes, 31 do not fall into ancient metazoan or bilaterian homeobox gene families; we deduce that they originated in the lophotrochozoan clade. We compared eight lophotrochozoan genomes to trace the pattern of homeobox gene evolution across this clade, allowing us to define 19 new lophotrochozoan-specific clades within the ANTP, PRD, TALE, ZF, SIX, and CUT classes. Using transcriptome data, we compared temporal expression of each homeobox gene in oyster development, and discovered that the lophotrochozoan-specific homeobox genes have peak expression either in early development (egg to gastrula) or in late development (after the trochophore larval stage), but rarely in between. This finding is consistent with the egg-timer, hourglass or phylotypic stage model of developmental evolution, in which there is a conserved central phase of development, but more evolutionarily labile early and late phases.

## Introduction

“The life of man is of no greater importance to the universe than that of an oyster*.*” David Hume (1775).

The Lophotrochozoa comprises approximately half of the phyla in the Animal Kingdom, including mollusks, annelids, platyhelminths, brachiopods, phoronids, bryozoans, and other phyla (Halanych et al. 1995). The lophotrochozoan clade is placed in the Bilateria, together with two other major groups showing bilateral symmetry, Ecdysozoa and Deuterostomia. Bilaterians include all animals with the exception of four phyla that descend from early diverging nodes in animal evolution (sponges, cnidarians, ctenophores, and placozoans). With a huge variety of body plans, there is no single morphological trait shared by all lophotrochozoans, hence their node-based definition (Halanych et al. 1995). The name is a composite of “lopho-,” derived from the lophophore, the feeding structure present in brachiopods, phoronids and bryozoans, and “-trochozoa,” based on a trochophore larva found in annelids, mollusks and others, although some lophotrochozoans possess neither of these traits (gastrotrichs, gnathostomulids, rotiferans, etc.). Other authors have suggested an alternative name, Spiralia, based on the view that this mode of embryonic cleavage was most likely present in the last common ancestor (LCA) of the group (Giribet 2002, 2008). Here we use the term Trochozoa to define a clade nested within Lophotrochozoa, including mollusks and annelids but excluding platyhelminths and rotifers. The Lophotrochozoa comprise a huge diversity of body types, developmental patterns, and life cycles, making them an ideal group to study the evolution of development.

The first lophotrochozoan genomes were published in 2009, those of the platyhelminths *Schistosoma mansoni* (Berriman et al. 2009) and *Schistosoma japonicum* (*Schistosoma japonicum* Genome Sequencing and Functional Analysis Consortium 2009), both parasitic trematodes. These were followed by two bivalve mollusk genomes, the Pacific oyster (Zhang et al. 2012) and the Akoya pearl oyster (Takeuchi et al. 2012), the genomes of a gastropod mollusk and two annelids (Simakov et al. 2012), the genomes of four further parasitic flatworms (Tsai et al. 2013; Zheng et al. 2013), and the genome of an asexual bdelloid rotifer (Flot et al. 2013). This plethora of genome sequences can now be used to examine the evolution of complex sets of genes, such as the large homeobox gene superclass that encodes transcription factors with regulatory functions in development. Homeobox genes of animals are divisible into 11 major classes defined through gene phylogenies and/or presence of additional domains in the encoded protein. The two largest gene classes are the ANTP class (including Hox genes plus many others) and the PRD class; other important developmentally expressed genes are also found in the TALE, POU, CUT, SINE, PROS, CERS, HNF, ZF, and LIM classes (Holland 2013). Gene classes are split into gene families comprising smaller sets of evolutionarily related genes; around 100 gene families can be traced to the LCA of the Bilateria, with some of these families dating back to earlier metazoan nodes (Srivastava et al. 2008, 2010; Ryan et al. 2010, 2006). Other gene families have more restricted phylogenetic distribution and presumably arose by duplication and extreme divergence from older homeobox genes, with their precise origin now obscured by sequence divergence. Examples of homeobox gene families discussed in this study include En, Hmx Pou1, Barx, Hopx, and Pax4/6. Homeobox genes have proven to be good markers to trace the major evolutionary changes in genomes; for example, they have been used as a proxy to assess evolutionary stasis in genomes (Paps et al. 2012), genome simplification (Tsai et al. 2013; Hahn et al. 2014), convergent evolution (Irimia et al. 2008, 2011; McGonnell et al. 2011; Maeso et al. 2012), the effects of asexuality and tetraploidy (Flot et al. 2013), and the impact of whole-genome duplications (Holland et al. 1994). Homeobox genes have been described in some of the lophotrochozoan genomes (Simakov et al. 2012; Flot et al. 2013; Morino et al. 2013; Tsai et al. 2013; Hahn et al. 2014), but thus far no attempt has been made to analyze them collectively to assess their evolution across the Lophotrochozoa.

Here we compare homeobox gene complements between eight lophotrochozoan genomes, plus representatives of other metazoans, together with a detailed analysis of all homeobox genes in the genome of the Pacific oyster. Our analyses support the classical gene families shared across bilaterians, but also reveal 19 lineage-specific homeobox gene groups found only within lophotrochozoans. These “novel” genes are of different ages, ranging from comparatively old genes dating to the base of the Lophotrochozoa, to more recent genes shared only by closely related species. Using transcriptome data for the Pacific oyster we find that the novel homeobox genes have peak expression either early or late in development, but rarely in the trochophore, implying that the intermediate temporal stages of lophotrochozoan development are comparatively stable evolutionarily and less able to accommodate new and divergent regulatory genes.

## Materials and Methods

### Assembly of Homeobox Data Sets

Homeobox sequences already identified in the following lophotrochozoan genomes were generously made available by their respective authors: Owl limpet *Lottia gigantea*, polychaete annelid *Capitella teleta,* and leech *Helobdella robusta* (Simakov et al. 2012), bdelloid rotifer *Adineta vaga* (Flot et al. 2013), and Akoya pearl oyster *Pinctada fucata* (Morino et al. 2013). Sequences from the cestode *Echinococcus granulosus* and the trematode *S. mansoni* were already identified by some of the authors (Tsai et al. 2013). The Florida amphioxus (*Branchiostoma floridae*) and the red flour beetle (*Tribolium castaneum*) were, respectively, used as representatives of deuterostomes and ecdysozoans; these sequences were recovered from the online resource HomeoDB2 (Zhong et al. 2008; Zhong and Holland 2011). Amphioxus and beetle were selected because their homeobox gene sequences are less divergent than other members of these groups (e.g., *Drosophila melanogaster*, *Caenorhabditis elegans*, and *Ciona intestinalis*), and they have not suffered whole-genome duplication events (such as in vertebrates); both factors that complicate orthology assignment. To recover as much taxonomic diversity as possible for the Lophotrochozoa, the Pfam database (Finn et al. 2010) was also mined for homeobox domains found in its representatives (applying a taxonomic restriction in the “species” tab of family record PF00046). Pfam only provides the sequence region belonging to the domain of interest, thus the complete sequences for these 941 lophotrochozoan homeobox genes were extracted from UniProt (Uniprot Consortium 2014). To avoid incomplete sequences, only UniProt records longer than 40 amino acids were kept, reducing the data set to 489 sequences; these were added to the homeobox genes of the complete genomes and HomeoDB indicated above.

To obtain all homeobox genes from the Pacific oyster genome, we used a strategy described previously (Marlétaz et al. 2014). Briefly, all lophotrochozoan Pfam homeodomains, plus those of amphioxus and beetle, were used as queries to perform local BLAST+ (Camacho et al. 2009) searches of the *Crassostrea gigas* predicted proteins and genome (EnsemblMetazoa GCA_000297895.1, assembly version 9.1); in parallel, a HMMER (Eddy 2009) search was performed using the “hmm” profile for the homeodomain provided by Pfam (PF00046). The lists of candidate predicted proteins from both BLAST and HMMER were merged and redundancies removed.

### Phylogenetic Analyses and Classification

**Fig. 1.—:**
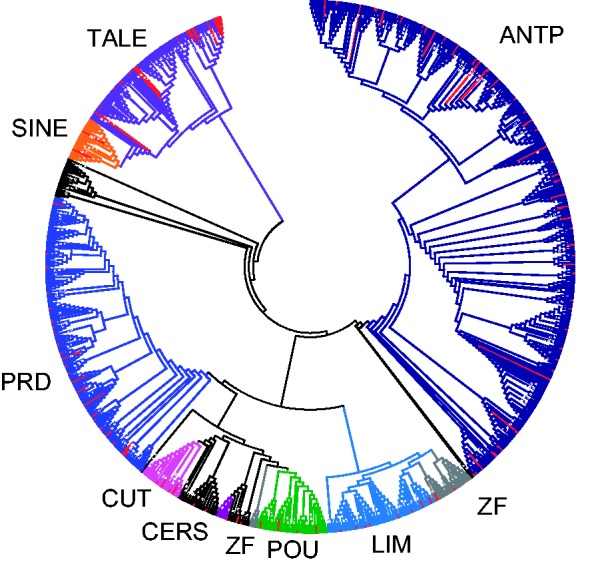
Cladogram displaying the diversity of homeobox genes. The tree, with 1,940 terminal branches, shows the evolution the homeobox gene complement of ten complete bilaterian genomes (eight lophotrochozoans, one ecdysozoan, and one deuterostome) and all the homeobox domains annotated for other lophotrochozoans in Pfam (see text). The interspersed red branches denote Pacific oyster sequences. The same tree, with branch lengths, support values and gene names, is presented in supplementary figure S1, Supplementary Material online.

Homeodomain proteins were aligned using MAFFT (Katoh et al. 2002) with the E-INS-I algorithm and checked in BioEdit (Hall 1999) to detect regions of ambiguous alignment. The resulting alignment belonging to the homeodomain region (1,940 sequences, supplementary file S1, Supplementary Material online) was used to perform maximum-likelihood phylogenetic analyses with the program RAxML (Stamatakis 2006) using the evolutionary model LG + Gamma + Invariant (Le and Gascuel 2008); 1,000 replicates were produced to obtain the bootstrap support (BS) values (fig. 1 and Supp fig. 1). A second data set was produced by removing sequences belonging to *H. robusta* and *A. vaga*, as both genomes have many divergent paralogs that were found to complicate phylogenetic inference; then this alignment was divided in subsamples, each containing one or few homeobox gene classes (supplementary figs. S2–S6, Supplementary Material online). To optimize gene classification for homeobox genes of *C. gigas,* we also used Conserved Domains Database to identify domains outside the homeodomain (Bürglin 2011), examined amino acid insertions/deletions in the homeodomain (i.e. TALE genes, HNF, *Cmp*,** or *Prox*), and diagnostic amino acids notably at position 50 of the homeodomain (K in SINE Class, *Gsc* and *Mix*, I/A/G in TALE genes, H in *Cux*) (Bürglin 2011; Marlétaz et al. 2014).

### Gene Expression Analyses

RNA-seq data, given as RPKM values (reads per kilobase per million reads) were from supplementary table S14 of Zhang et al. (2012), mapped to automated gene predictions apart from *Hox5, Mnx2, Gsc3, Prox, Zeb, Hbx3*,** and *Hbx4* which were not originally predicted. A heat map of gene expression was drawn normalizing the gene expression for each gene and sorting the genes by their peak of expression.

## Results

### Diversity of Homeobox Genes in the Pacific Oyster

We identified and classified 136 homeobox genes in the previously reported genome sequence of the Pacific oyster *C. gigas* (Zhang et al. 2012), using a combination of phylogenetic analysis, sequence identity, domain composition, and specific molecular traits (supplementary table S1, Supplementary Material online). The phylogenetic analysis was performed on a large data set comprising 1,940 sequences, including all known lophotrochozoan homeobox genes (fig. 1 and supplementary fig. S1, Supplementary Material online; alignment available as supplementary file S1, Supplementary Material online), and also on subsets of the data (supplementary figs. S2–S6, Supplementary Material online). All analyses produced the same overall topology, recovering monophyly of the main homeobox gene classes with few exceptions (TALE, ZF, and PRD in the largest data set, fig. 1). Companion domains, motifs, and insertions/deletions specific to certain gene families/classes were also examined and found to be congruent with the position of the sequences in the gene trees (Paired domain in PRD genes, Tinman motif in NK genes, 3-amino acid insertion in TALE genes, etc). Deviations from monophyly are likely to be due to limited phylogenetic signal present in the 60 amino acids of the homeodomain, the only region alignable across the full data set, combined with a high number of sequences that can increase homoplasy within the data set. The analyses using subsets of the data, containing one or few homeobox classes at a time, provided trees with a higher number of well-resolved nodes; these form the basis of the classification described below.

The Pacific oyster has genes in 10 of the 11 metazoan homeobox classes (tables 1 and 2); the class missing is HNF, discussed below in the context of Lophotrochozoa evolution. When classifying these genes, we identified duplications of previously known genes (*En* and *Hmx*), plus 31 homeobox genes that do not have clear orthologs in the sequenced genomes of ecdysozoans and deuterostomes, and cannot therefore be assigned to known gene families. These are discussed later.

**Table 1:** Oyster Homeobox Genes

Classes	Gene Family	Gene Name	Gene Model
ANTP	Cdx	*Cdx*	CGI_10023003
	Evx	*Evx1*	CGI_10013056
	Evx	*Evx2*	CGI_10013058
	Gbx	*Gbx*	CGI_10012203
	Gsx	*Gsx*	CGI_10015548
	Hox1	*Hox1*	CGI_10024083
	Hox2	*Hox2*	CGI_10024086
	Hox3	*Hox3*	CGI_10024087
	Hox4	*Hox4*	CGI_10024091
	Hox5	*Hox5*	scaffold801_482925 _483164
	Hox6-8	*Lox5*	CGI_10026565
	Hox6-8	*Lox2*	CGI_10018592
	Hox6-8	*Lox4*	CGI_10026562
	Hox9-13(15)	*Post1*	CGI_10027385
	Hox9-13(15)	*Post2*	CGI_10027388
	Meox	*Hrox*	CGI_10014888
	Mnx	*Mnx1*	CGI_10026625
	Mnx	*Mnx2*	scaffold313_810611 _810757
	Pdx	*Xlox*	CGI_10015546
	Barhl	*BarH1*	CGI_10009941
	Barhl	*BarH2*	CGI_10009942
	Barhl	*BarH3*	CGI_10009943
	Barx	*Barx*	CGI_10004014
	Bsx	*Bsx*	CGI_10008107
	Dbx	*Dbx*	CGI_10002480
	Dlx	*Dlx*	CGI_10016653
	Emx	*Emx1*	CGI_10018603
	Emx	*Emx2*	CGI_10025052
	Emx	*Emx3*	CGI_10025053
	En	*En1*	CGI_10012208
	En	*En2*	CGI_10012209
	Hbn	*Hbn*	CGI_10011181
	Hhex	*Hex*	CGI_10025054
	Hlx	*Hlx*	CGI_10013972
	Lbx	*Lbx*	CGI_10010398
	Msx	*Msx*	CGI_10023979
	Msxlx	*Msxlx*	CGI_10013606
	Nk1	*Nk1*	CGI_10025189
	Nk2.1	*Nkx2.1*	CGI_10021129
	Nk2.2	*Nk2.2*	CGI_10026839
	Nk3	*Nk3*	CGI_10023919
	Nk4	*Nk4*	CGI_10019417
	Nk5/Hmx	*Hmx1*	CGI_10013448
	Nk5/Hmx	*Hmx2*	CGI_10027035
	Nk6	*Nk6*	CGI_10028825
	Nk7	*Nk7*	CGI_10027184
	Noto	*Noto*	CGI_10013404
	Ro	*Ro*	CGI_10005958
	Tlx	*Tlx1*	CGI_10020596
	Tlx	*Tlx2*	CGI_10020599
	Vax	*Vax*	CGI_10020700
	ANTP_NKL Clade I	*Cgi_NKL*	CGI_10028802
PRD	Arx	*Arx*	CGI_10028810
	Dmbx	*Dmbx*	CGI_10011833
	Drgx	*Drgx*	CGI_10007626
	Gsc	*Gsc1*	CGI_10007832
	Gsc	*Gsc2*	CGI_10026711
	Gsc	*Gsc3*	scaffold42840_31020 _31181
	Hopx	*Hopx*	CGI_10009529
	Otp	*Otp*	CGI_10021751
	Otx	*Otx*	CGI_10015784
	Pax3/7	*Pax3/7*	CGI_10026438
	Pax4/6	*Pax4/6*	CGI_10020873
	Pax4/6	*Pax6*	CGI_10027695
	Pitx	*Pitx*	CGI_10018398
	Prop	*Prop*	CGI_10006125
	Prrx	*Prxx*	CGI_10021523
	Rax	*Rax*	CGI_10028663
	Repo	*Repo*	CGI_10005826
	Shox	*Shox*	CGI_10012343
	Uncx	*Uncx*	CGI_10007529
	Vsx	*Vsx1*	CGI_10010562
	Vsx	*Vsx2*	CGI_10010563
	PRD Clade IV	*Cgi_PRD1*	CGI_10017003
	n.d.	*Cgi_PRD2*	CGI_10012650
	PRD Clade VI	*Cgi_PRD3*	CGI_10009720
	PRD Clade V	*Cgi_PRD4*	CGI_10003333
	PRD Clade V	*Cgi_PRD5*	CGI_10013213
	PRD Clade I	*Cgi_PRD6*	CGI_10015407
	PRD Clade III	*Cgi_PRD7*	CGI_10025814
	PRD Clade II	*Cgi_PRD8*	CGI_10026008
	n.d.	*Cgi_PRD9*	CGI_10026078
LIM	Isl	*Islet*	CGI_10028355
	Lhx1/5	*Lhx1/5*	CGI_10025343
	Lhx2/9	*Lhx2/9*	CGI_10015423
	Lhx3/4	*Lhx3/4*	CGI_10028171
	Lhx6/8	*Awh1*	CGI_10025669
	Lhx6/8	*Awh2*	CGI_10020871
	Lmx	*Lmx1*	CGI_10019449
	Lmx	*Lmx2*	CGI_10019450
POU	Pou2	*Pou2*	CGI_10006547
	Pou3	*Pou3*	CGI_10005968
	Pou4	*Pou4*	CGI_10023764
	Pou6	*Pou6*	CGI_10028901
SINE	Six1/2	*Six1/2*	CGI_10009922
	Six3/6	*Six3/6*	CGI_10027570
	Six4/5	*Six4/5*	CGI_10022945
TALE	Irx	*Irx1*	CGI_10011883
	Irx	*Irx2*	CGI_10028533
	Irx	*Irx3*	CGI_10020098
	Irx	*Irx4*	CGI_10004700
	Meis	*Meis*	CGI_10019589
	Mkx	*Mkx*	CGI_10011802
	Pbx	*Pbx*	CGI_10026001
	Pknox	*Pknox*	CGI_10011944
	Tgif	*Tgif*	CGI_10023491
	TALE Clade II	*Cgi_TALE1*	CGI_10000035
	TALE Clade I	*Cgi_TALE2*	CGI_10008335
	TALE Clade III	*Cgi_TALE3*	CGI_10011283
	TALE Clade VII	*Cgi_TALE4*	CGI_10013112
	n.d.	*Cgi_TALE5*	CGI_10014640
	TALE Clade V	*Cgi_TALE6*	CGI_10019516
	TALE Clade IV	*Cgi_TALE7*	CGI_10015054
	TALE Clade IV	*Cgi_TALE8*	CGI_10015055
	TALE Clade VI	*Cgi_TALE9*	CGI_10015742
	TALE Clade VI	*Cgi_TALE10*	CGI_10019518
	TALE Clade VI	*Cgi_TALE11*	CGI_10021477
	TALE Clade VI	*Cgi_TALE12*	CGI_10021478
	TALE Clade VI	*Cgi_TALE13*	CGI_10015317
	TALE Clade IV	*Cgi_TALE14*	CGI_10021576
CUT	Cmp	*Cmp1*	CGI_10015221
	Cmp	*Cmp2*	CGI_10015220
	Cux	*Cux*	CGI_10022123
	Onecut	*Onecut*	CGI_10019668
	Cut Clade I	*Cgi_CUT1*	CGI_10006727
	Cut Clade I	*Cgi_CUT2*	CGI_10022104
PROS	Prox	*Prox*	CGI_10022026
ZF	Zeb	*Zeb*	scaffold42570_54922 _55110
	Zfhx	*Zfhx*	CGI_10012804
	Zhx/Homez	*Homez*	CGI_10009971
	n.d.	*Cgi_ZF*	CGI_10018873
CERS	Cers	*Cers*	CGI_10021077
Others	n.d.	*Cgi_Hbx_1*	CGI_10004542
	n.d.	*Cgi_Hbx_2*	CGI_10016179
	n.d.	*Cgi_Hbx_3*	CGI_10007234
	n.d.	*Cgi_Hbx_4*	scaffold1627_218521 _218706

Note.—The homeobox gene complement of *Crassostrea gigas,* classified into gene classes and gene families. Protein models for each gene are indicated. n.d. denotes that the gene family cannot be determined, usually due to divergence of the homeodomain sequence and presence only in oyster.

**Table 2:** Oyster Homeobox Complement Compared with Other Bilaterians

Homeobox	*Crassotrea gigas*	*Drosophila melanogaster*	*Strongylocentrotus purpuratus*	*Branchiostoma floridae*	*Homo sapiens*
Number of genes	136	104	97	133	255
ANTP	53	47	38	60	100
PRD	30	28	32	29	66
LIM	8	6	6	7	12
POU	4	5	4	7	16
HNF	0	0	2	4	3
SINE	3	3	3	3	6
TALE	23	8	6	9	20
CUT	6	3	1	4	7
PROS	1	1	1	1	2
ZF	4	2	3	5	14
CERS	1	1	1	1	5
Other	4	0	0	3	4

Note.—Distribution of oyster genes in homeobox classes compared with some well-characterized bilaterians (Zhong and Holland 2011).

### Chromosomal Organization of Pacific Oyster ANTP Class Genes

**Fig. 2.—:**
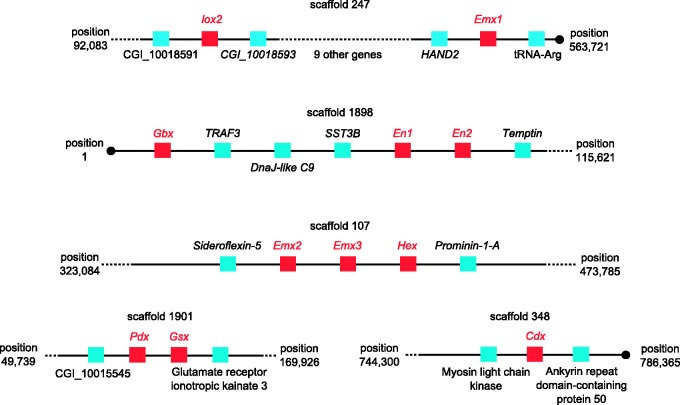
Physical clustering of oyster ANTP class genes, excluding Hox genes. Most ANTP class genes show no clustering in the oyster genome. Linkages found between ANTP genes (in red) are shown for five scaffolds of Pacific oyster genome assembly version 9.1. Scaffolds are not represented to scale, numbers indicate the nucleotide positions defining each genomic region within a scaffold. When the genes shown are not located at the end of a scaffold, no other homeobox genes were found for the next five gene models. Dashed lines indicate the presence of additional genes in the scaffold; black circles denote the end of scaffolds.

The ANTP class is thought to have expanded by tandem gene duplication in early metazoan evolution to generate a large array of linked genes; these split into at least four chromosomal regions: The SuperHox cluster (Hox genes plus many linked homeobox genes [Butts et al. 2008]), the ParaHox gene cluster (Brooke et al. 1998), the NK-linked (NKL) array, and the NK2.1/2.2 pair (Holland 2013). Within these chromosomal regions some (but not all) animal taxa have retained tight clustering of certain genes (notably the Hox cluster, ParaHox cluster, NK cluster, and *NK2.1/2.2* pair). We examined scaffolds from the Pacific oyster genome assembly version 9.1 to determine which ANTP class genes were linked or clustered (table 1; fig. 2). We found that oyster genes in the ANTP gene class show an unusually low extent of clustering, with extensive scattering of genes onto distinct scaffolds. The breakage of the oyster Hox gene cluster into four regions has already been reported (Zhang et al. 2012). Within the SuperHox genes, we find that the pair of *Evx* genes is not found close to Hox genes, although one *Emx* gene (*Emx1*) is on the same scaffold as one Hox gene (*Lox2*, fig. 2). Three other SuperHox genes (*En1, En2*,** and *Gbx*) are neighbors of each other. Scattering is also seen for the NKL genes, with little evidence for retention of clustering or pairs of genes. The *NK4* and *NK3* genes, located together in many taxa, are separate, as are the *Tlx* and *Lbx* genes. We find a multiplication of linked *BarH* genes and tandem duplications of *Tlx* and *Emx*, but no other cases of multiple NKL genes on the same scaffold. We do find one scaffold with representatives of both the SuperHox group and the NKL group: The *Hex* gene (SuperHox) is directly adjacent to the *Emx2* and *Emx3* (NKL). Turning to ParaHox genes, *Pdx* and *Gsx* form a closely linked pair, consistent with their origin from the ParaHox gene cluster, but the third ParaHox gene *Cdx* is on a different scaffold.

### Origin and Loss of Ancient Bilaterian Homeobox Gene Families

**Fig. 3.—:**
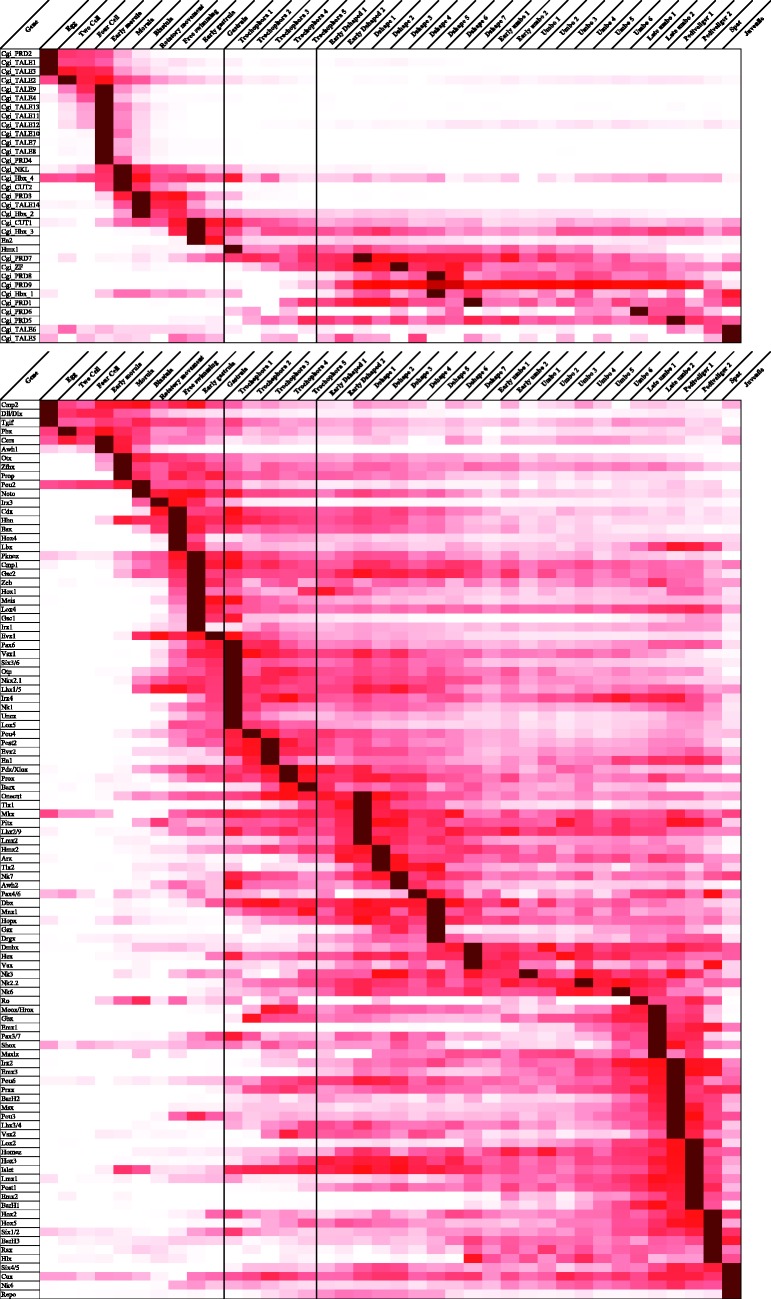
Heatmap. Heat map representing the temporal expression patterns of oyster homeobox genes. Top panel shows genes belonging to clades found only in the Lophotrochozoa; bottom panel shows genes shared with other bilaterian superclades. The trochophore larval stage is outlined with back lines.

Increasing taxon sampling can radically alter inferences about origin and loss of any trait in biology. We compiled and analyzed a data set of approximately 2,000 bilaterian homeobox sequences, with particular emphasis on lophotrochozoan species. This broad sampling pushes back the date of origin of some homeobox gene families and highlights gene loss in others. Key patterns include as follows:

- The homeobox gene family *Barx* (ANTP class), previously known only from deuterostomes, is found in several lophotrochozoans (three mollusks, the rotifer, and one annelid; supplementary fig. S1, Supplementary Material online). This pushes its date of origin back to the base of Bilateria.
- The homeobox gene family *Hopx* family (PRD class), thus far only reported from Chordata, is found in mollusks (100% BS for the sequences found in the two oysters, 67% for all together including snail; supplementary fig. S3, Supplementary Material online). This pushes its date of origin back to the base of Bilateria and suggests multiple *Hopx* gene losses in bilaterian evolution.
- The class HNF was previously known only from the cnidarian *Nematostella vectensis* (Ryan et al. 2006), the nematode *Ca. elegans,* and deuterostomes (Howard-Ashby et al. 2006; Zhong and Holland 2011). Thus, it was presumed lost in lophotrochozoans. However, we find tentative evidence for HNF sequences in the rotifer and the two annelids (*Tcf1*, 50% in supplementary fig. S4, Supplementary Material online) but not in the Pacific oyster. Two of the annelid sequences show an indel between helix 2 and 3, as seen in *Hnf* homeodomain of other metazoans, although the length and sequence are different to that encoded by amphioxus *Hnf* genes.
- The *Pou1* gene family, formerly only known from nonbilaterians and deuterostomes, is now tentatively found in three annelids and one flatworm (47% in supplementary fig. S6, Supplementary Material online). There are other annelid and flatworm sequences representing each of the other *Pou* gene families; this increases the likelihood that the tentative assignment to the *Pou1* clade is correct. These results suggest that *Pou1* was not lost once in the common ancestor of protostomes, but instead it was lost (at least) in an ancestor of Ecdysozoa and multiple times within Lophotrochozoa.
- The homeobox *Zeb* gene (ZF class) shows a patchy distribution, with a putative ortholog found in limpet (39%; supplementary fig. S5, Supplementary Material online), along with the known distribution in deuterostomes, the nematode *C. elegans*, beetle *Tribolium* and *Drosophila zfh1* (Zhong and Holland 2011). However, ZF genes are difficult to classify and their taxonomic distribution should be interpreted cautiously.
- The *En* (engrailed) homeobox gene family (ANTP class) shows multiple paralogs in Lophotrochozoa (supplementary figs. S1 and S2, Supplementary Material online). Our analyses group one *En* paralog with the *En* gene in other bilaterians; the second copy is only found in members of the lophotrochozoan clade. Taken at face value this could imply an early duplication and loss in Ecdysozoa and Deuterostomia; however, the most parsimonious scenario is for this gene being a lophotrochozoan-specific gene duplication with sequence divergence of one duplicate. Lack of resolution in the gene trees does not clarify the relationships between the lophotrochozoan-specific *En* paralogs. When the expression levels are compared in *C. gigas* (fig. 3), *en1* was found to have low, homogeneous expression across developmental stages, whereas the lophotrochozoan-specific gene (*en2*) has a peak of expression in the early gastrula. *En* has been previously linked to the formation of shell in a snail (Moshel et al. 1998), and the oyster *en2* paralog has been shown to peak its expression at the same time as the shell gland appears in the embryo (Zhang et al. 2012).
- As described elsewhere, there are massive gene family losses in the parasitic Platyhelminthes (Tsai et al. 2013; Hahn et al. 2014), including 24 homeobox gene family losses shared by all parasitic flatworms, several lineage-specific losses as well as three convergent homeobox gene losses between monogeneans and cestodes, and four between monogeneans and trematodes (Hahn et al. 2014).
- As previously shown (Simakov et al. 2012), the leech *H. robusta* has an unusually expanded homeobox gene complement (181 genes) including 14 paralogs of the ParaHox gene *Cdx* and three copies of *Hox5*.
- It was already reported that many gene families that are single copy in most species are in two or more copies in the rotifer *A. vaga*, for example up to eight duplicates of *Pax 4/6* are found (Flot et al. 2013). This is consistent with the possible tetraploid genome of this asexual bdelloid rotifer.

### Evolution and Expression of Novel Homeobox Gene Families in Lophotrochozoa

Performing phylogenetic analysis using all oyster homeobox genes, plus homeobox genes of additional lophotrochozoans and outgroups, allows us to define 17 new homeobox gene clades in Lophotrochozoa (table 3, supplementary fig. S7 and table S2, Supplementary Material online). These clades include NKL Clade I, PRD Clades I–VI, TALE Clades I–VII, and CUT Clade I (each with oyster representatives). In addition to their position in gene trees, in some cases the Pacific oyster sequences belonging to these new clades hold molecular signatures relating them to these classes or families (supplementary table S1, Supplementary Material online): *Cgi_PRD1* has a paired domain (the other novel oyster PRD genes do not), new TALE genes display the characteristic 3-amino acid indel, of this class and there is a CUT domain present in *Cgi_Cux1* (but not in *Cgi_Cux2*). We also find lophotrochozoan-specific clades in the SIX and LIM classes, without oyster representatives. On top of these novel clades, the *En* and *Hmx* gene families also show duplicate genes taxonomically restricted to the Lophotrochozoa; all the lophotrochozoan-specific *En* sequences share a conserved cysteine in the position 23 of the homeodomain, and the two mollusk-specific *Hmx* genes share a histidine in position 10 (supplementary fig. S7, Supplementary Material online). Some species, including oyster, also have a few additional homeobox genes that do not group with genes of other species, and are currently considered orphans. We are not including two lophotrochozoan-specific clades of genes reported in a previous study (supplementary fig. S4.6.1 in Simakov et al. 2012), as in our analyses these are not recovered presumably due to the increased taxon sampling; we recover the protostome-specific CG11294 clade described in that study, including now the oyster gene Cgi PRD7, and name this clade PRD Clade III.

**Table 3:** Novel Homeobox Clades in the Lophotrochozoa

Clades		Taxonomic distribution	Support (%)
ANTP_NKL	Clade I	Trochozoa	27
ANTP_NKL	Hmx	Bivalvia	98
ANTP_NKL	engrailed	Lophotrochozoa	—
PRD	Clade I	Trochozoa	67
PRD	Clade II	Trochozoa	82
PRD	Clade III	Protostomes	12
PRD	Clade IV	Trochozoa	39
PRD	Clade V	Lophotrochozoa	—
PRD	Clade VI	Trochozoa	—
LIM	Clade I	Platyhelminthes Neodermata	86
SIX	Clade I	Platyhelminthes Neodermata	30
CUT	Clade I	Lophotrochozoa	—
TALE	Clade I	Trochozoa	99
TALE	Clade II	Bivalvia	44
TALE	Clade III	Lophotrochozoa	10
TALE	Clade IV	Lophotrochozoa	14
TALE	Clade V	Bivalvia	93
TALE	Clade VI	Mollusca	—
TALE	Clade VII	Bivalvia	26

Note.—List of the lophotrochozoan-specific homeobox gene clades found and their phylogenetic origin. Clades show varying levels of statistical support in gene trees. This list also includes a clade CG11294 defined in a previous study (Simakov et al. 2012), here named PRD Clade III. “—” indicate putative groups without monophyletic support.

Although some of the putative clades we define have low levels of support in phylogenetic trees (table 3), and some comprise a small number of genes (supplementary fig. S7, Supplementary Material online), we detect some short conserved (or semiconserved) motifs that give further confidence in these groupings of genes. These include the amino acids KEKR at the C-terminus of ANTP NK Clade I, SPQQVRS within the sequence of PRD Clade VI, and QVKK found within TALE Clade VI. The uncertainties for some clades prevent their erection as formal gene families, term we reserve for gene groups with a well-defined evolutionary history. Independent of the evolutionary relationships among these new genes, we are confident these are novel homeobox genes only found in the Lophotrochozoa.

The origin of the lophotrochozoan-specific groups can be dated to different points in phylogeny. Six clades can be traced back to the LCA of Lophotrochozoa (TALE Clades III and IV, PRD Clade V, and CUT Clade I); this is in addition to the previously mentioned duplication of *En*. Four clades were present in the LCA of Annelida plus Mollusca (ANTP NKL Clade I, PRD Clades I, II, IV, and VI, TALE Clade I), but due to the high levels of gene loss in the genomes of flatworms (Tsai et al. 2013; Hahn et al. 2014) and the rotifer (Flot et al. 2013) an earlier origin cannot be discounted. Two clades are shared only by the Trematoda and Cestoda (SIX Clade I and LIM Clade I), one clade is restricted to Mollusca (TALE Clade VI), and three groups are shared only by the two bivalve species analyzed (TALE Clade II, V, and VII; in addition to a duplication of the Hmx gene). No large companion domains were found in the novel-deduced homeodomain proteins (supplementary table S1, Supplementary Material online), with the exception of an approximately 87-amino acid conserved region N-terminal to the homeodomain of the molluscan-specific TALE Clades VI and VII (positions 289–376 in supplementary fig. S8, Supplementary Material online). We call this the PADRE domain based on a sequence in three of the proteins, and suggest that it may act as a functional domain in these proteins. The Pacific oyster has 25 genes within the 19 lophotrochozoan-specific clades (tables 1 and 3).

As these novel clades have no apparent counterparts in other animal taxa, we deduce that they have originated by duplication from older homeobox genes. After duplication, they have diverged such that they form distinct clades in phylogenetic analysis and their relationship to other families is now obscured. Following accepted nomenclature practice, we therefore consider them as essentially novel genes. The origin of new regulatory genes offers the opportunity to examine which developmental stages are more or less prone to evolutionary modification. Developmental stages that are tightly constrained would be expected to be less tolerant to the integration of new genes into their regulatory gene networks. We therefore wished to determine the developmental stages at which each novel homeobox gene was expressed.

**Fig. 4.—:**
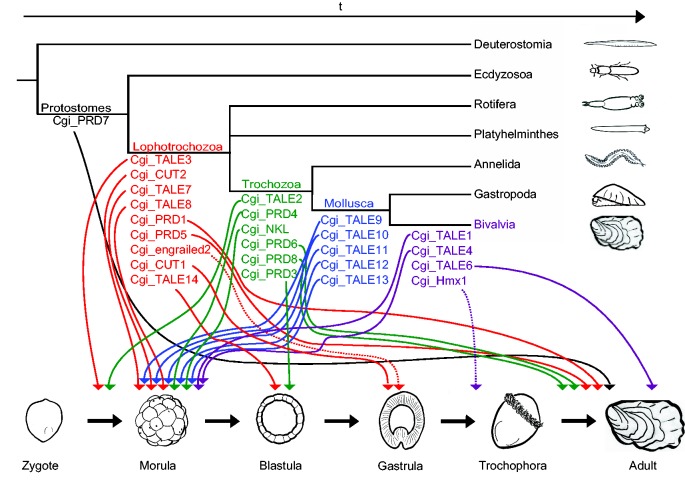
Phylogeny and ontogeny of the novel genes. Temporal expression peaks of different novel homeobox genes in relation to their evolutionary ages. Most phylogenetic nodes display genes with expression before and after the trochophore larvae stage; this stage seems most resilient to the addition of new homeobox gene expression.

As part of a previous study, we generated transcriptome data from a developmental time series of the Pacific oyster (Zhang et al. 2012). Here we used these data to determine the expression levels of all homeobox genes in the Pacific oyster, and compared each gene using gene expression temporal patterns (fig. 3). A striking finding is that several of the novel homeobox genes have similar expression patterns and cluster together based on temporal expression profile. This is particularly noticeable at the earliest stages of development (egg to blastula), when we find all but two of the novel TALE class genes expressed in oyster (genes *TALE1*–*TALE4* and *TALE7*–*TALE14*). The novel *PRD2* and *PRD4* genes are also expressed at this time. Most of the novel genes expressed after the trochophore larvae stage belong to the PRD class (*PRD1, 5, 6, 7, 8*), in addition to *TALE5* and *TALE6*. Both sets, early and late expressed, include genes that arose at various times in lophotrochozoan evolution. Very few other homeobox genes in oyster, especially the ancient genes conserved across Bilateria, share this unusual temporal expression profile (fig. 3). When we take lophotrochozoan-specific homeobox genes only, and plot their peak of expression across a developmental series, we see this early peak of expression very clearly (figs. 3 and 4). We also detect a second peak, for different lophotrochozoan-specific homeobox genes, in late development after the trochophore stage (figs. 3 and 4). We do not see any distinctly novel gene expressed at an intermediate developmental stage: Novel genes are recruited to either early development or late development. The only exception is the lophotrochozoan-specific *Hmx1* gene duplicate, an expansion within a gene family and not a highly divergent gene. This expression at such a critical developmental stage of lophotrochozoans may indicate an important role for *Hmx1* in the evolution of the superclade. To test whether the temporal patterns of expression are different between older and newer genes, we defined three temporal categories (early, trochophore, late) and compared the number of genes peaking at each stage. The older homeobox genes show a mixture of peak expression times (27% early, 17% trochophore, 56% late). In contrast, the younger genes show a significantly different distribution of peak expression times (chi-square *P* < 0.001), with 67% peaking early, 3% at trochophore (Hmx1), and 30% later in development. Although newer genes seem more prone to peak in early and late development, the trochophore stage seems relatively refractory to incorporating expression of novel homeobox genes.

## Discussion

The number of predicted homeobox genes for the Pacific oyster, 136, is higher than in some well-studied invertebrates (e.g., 104 genes in fruit fly and 91 in the honeybee; table 2) but not dissimilar from amphioxus (133 genes) and far lower than the number of homeobox genes in vertebrates (e.g., more than 250 genes in humans, table 2) (Zhong and Holland 2011). There are caveats to the precise number of homeobox genes, since as with the majority of genome sequences there are small gaps between sequenced contigs that may hold genes, and because assembly methods may artifactually merge closely related genes or separate distinct alleles. Nonetheless, this figure is expected to be close to the correct value for the Pacific oyster.

Understanding the evolution of these genes is helped greatly by comparison to other genomes, including additional published lophotrochozoan genomes. For example, the increased taxon sampling revealed that *Barx* and *Hopx* genes are older than formerly thought. Perhaps the most striking finding from the comparative study reported here is the large number of lophotrochozoan-specific homeobox genes: Among the 136 putative Pacific oyster homeobox genes, 31 genes do not have clear orthologs outside the Lophotrochozoa. Although genes can evolve from nongenic DNA (Carvunis et al. 2012), all genes in this study possess a recognizable homeobox. We therefore deduce that they originated by duplication from more ancient homeobox genes, but have undergone sufficient divergence that their origins are now obscured. Technically they are “cryptic paralogs” of older genes, but pragmatically they may be considered “new” or novel genes in view of the extensive sequence divergence. Highly divergent novel homeobox genes have been identified in other settings (e.g., amphioxus, human; Holland et al. 2007; Takatori et al. 2008) and in most cases the progenitors are unknown. In a few cases, such as *bicoid* gene of cyclorrhaphan flies and the Shx genes of Lepidoptera, the progenitor can be deduced (*zen*), but in these cases the evolutionary origin was only possible because of the rather unusual genomic organization of the Hox cluster and extensive data from closely related species (Stauber et al. 1999; Ferguson et al. 2014).

The lophotrochozoan-specific genes are sufficiently divergent from other homeobox genes to suggest that they are likely to have taken up novel roles, and quite possibly they regulate downstream target genes that are different from the targets of their cryptic progenitors. It is therefore interesting to determine the developmental processes or pathways into which they have been integrated. We do not know the spatial expression patterns of these genes, but we do find striking temporal expression patterns. In all cases, the divergent lophotrochozoan homeobox genes are expressed either in very early or in rather late developmental stages (fig. 4). This suggests that gene regulatory networks acting early and late in molluscan development have been modified by incorporation of new transcription factors, but the “middle” stages (notably in the trochophore) have not been subject to the same modification. Recruitment of new genes to very early development is paradoxical, as early cleavage stages are morphologically highly conserved between trochozoans with spiral cleavage. The lack of recruitment of new genes to the middle stages of development suggests that these stages may be the most resistant to evolutionary change. This finding is consistent with an emerging concept of greater evolutionary change in early and late developmental stages of animals. The most conserved period, typical for a given clade, is called the phylotypic stage (or period) and the overall pattern is referred to as the developmental egg-timer or hourglass (Duboule 1994; Sander 1994; Raff 1996). The pattern may reflect the existence of a stage in development when there is a greater constraint to evolutionary change, perhaps due to deployment of conserved patterning genes such as Hox genes and others (Slack et al. 1993; Duboule 1994; Sander 1994; Raff 1996). In recent years, several studies have been published in support of this contention, by demonstrating that a similar pattern extends to the molecular level. For example, the pattern is seen, albeit quite subtly, in the variation in gene expression between different species of *Drosophila* (Kalinka et al. 2010) or *Caenorhabditis* (Levin et al. 2012), in the temporal deployment of new genes in the phylogenetic history of insects (Domazet-Lošo and Tautz 2010) and in alterations to transcriptome complexity in vertebrate evolution (Irie and Kuratani 2011). Our results extend this general conclusion to molluscan evolution, and with a more striking signal than in previous studies. We suggest that this difference is because we focussed on genes encoding putative transcription factors, whereas other studies have analyzed global gene expression patterns. We suggest that the trochophore stage could be the phylotypic stage for mollusks; this hypothesis needs further testing using data from other taxa.

## Conclusions

We classify 136 homeobox genes in the genome of the Pacific oyster and compare with the homeobox gene complements of seven other lophotrochozoans, and other metazoans. We find that ANTP class homeobox genes show a low degree of clustering in the oyster genome. We also identify 25 oyster genes that most likely evolved within the lophotrochozoan lineage, and together with data from other taxa we define 19 lophotrochozoan-specific clades of homeobox genes. In oyster development, lophotrochozoan-specific genes have been recruited to early and late stages of development, supporting the egg-timer or hourglass model of developmental evolution.

## Supplementary Material

Supplementary material S1, figures S1–S9, and tables S1 and S2 are available at *Genome Biology and Evolution* online (http://www.gbe.oxfordjournals.org/).

Supplementary Data
