# Supplementary material for: Reinforcing the Egg-Timer: Recruitment of Novel Lophotrochozoa Homeobox Genes to Early and Late Development in the Pacific Oyster
Source: Genome Biol Evol. 2015 Jan 27;7(3):677–88. doi: 10.1093/gbe/evv018 (PMC5322547; doi:10.1093/gbe/evv018)
Supplement: Supplementary Data [file supp_7_3_677__index.html]

Reinforcing the Egg-Timer: Recruitment of Novel Lophotrochozoa Homeobox Genes to Early and Late Development in the Pacific Oyster — Supplementary Data 

# Reinforcing the Egg-Timer: Recruitment of Novel Lophotrochozoa Homeobox Genes to Early and Late Development in the Pacific Oyster

## Supplementary Data

files

**Files in this Data Supplement:**

- Supplementary Data - zip file
