# Supplementary material for: Reinforcing the Egg-Timer: Recruitment of Novel Lophotrochozoa Homeobox Genes to Early and Late Development in the Pacific Oyster
Source: Genome Biol Evol. 2015 Jan 27;7(3):677–88. doi: 10.1093/gbe/evv018 (PMC5322547; doi:10.1093/gbe/evv018)
Supplement: Supplementary Data [file supp_evv018_suppl_data.zip › Supp_Figure_7_Novel_homeobox_genes_alignments.pdf]

ANTP genes

```
                                              10        20        30        40        50
                                     ....|....|....|....|....|....|....|....|....|....|....|...
ANTP_NKL_Clade_en_Cgi_En2            KRPRTAFSNEQLQRLKVEFEK-CQYLTEQRRLELAKTLNLSEGQIKIWFQNKRAKVKK
ANTP_NKL_Clade_en_Euprymna_sco       -------TSDQLLRLKQEFDD-CRYLTEDRRKKLARELSLSEAQIKIWFQNKRAKIKK
ANTP_NKL_Clade_en_Graptacme_eb       QAXXXXXSNEQLDRLQVEFEA-CRYLTETRRKDLSDELKLSETQIKIWFQNKRAKLKK
ANTP_NKL_Clade_en_Haliotis_asi       KRPRTAFTNDQLQRLKKEFDD-CRYLTEQRRKDLALSLSLTEAQIKIWFQNKRAKIKK
ANTP_NKL_Clade_en_Ilyanassa_ob       QAXXXXXTSEQLSRLKREFDE-CRYLTETRRRHLAAELGLTESQIKIWFQNKRAKIKK
ANTP_NKL_Clade_en_Ilyanassa_ob       QAXXXXXTSEQLSRLKREFEE-CRYLTETRRRHLAAELGLTESQIKIWFQNKRAKIKK
ANTP_NKL_Clade_en_Ilyanassa_ob       -------TSEQLSRLKREFDE-CRYLTETRRRHLAAELGLTESQIKIWFQNKRAKIKK
ANTP_NKL_Clade_en_Lepidochiton       QAXXXXXSDSQLERLKKEFDS-SRYLSEAKRQDLARELRLNESQIKIWFQNKRAKLKK
ANTP_NKL_Clade_en_Lgig_1414609       KRPRVAFTNEQLEKLKVEFEE-CRYLTEARRRDLAIRLHLSESQIKIWFQNKRAKLKK
ANTP_NKL_Clade_en_Loligo_vulga       -------TSDQLQRLKQEFDD-CRYLTEERRKKLARELCLSEAQIKIWFQNKRAKIKK
ANTP_NKL_Clade_en_Lymnaea_stag       KRPRTAFTNDQLQRLKREFDE-CRYLTETRRKNLADELGLTESQIKIWFQNKRAKIKK
ANTP_NKL_Clade_en_Nautilus_pom       QAXXXXXTSDQLQYLKKEFEE-CRYLTEDRRRKLARELSLSEAQIKIWFQNKRAKMKK
ANTP_NKL_Clade_en_Pfuc_307148_       -------------MLKKEFET-CRYLTEQRRNQLSRDLDLSESQIKIWFQNKRAKIKK
ANTP_NKL_Clade_en_Pinctada_fuc       KRPRTAFSNDQLQRLKREFDS-CQYLTEQRRRDLARELQLSEGQIKIWFQNKRAKVKK
ANTP_NKL_Clade_en_Placopecten_       QAXXXXXTNDQLQRLKREFEE-CRYLTEQRRLDLAQELNLTEAQIKIWFQNKRAKIKK
Beetle_En_En_ANTP                    KRPRTAFSSAQLARLKHEFNE-NRYLTERRRQQLSAELGLNEAQIKIWFQNKRAKIKK
Beetle_Inv_En_ANTP                   KRPRTAFSGAQLARLKHEFAE-NRYLTERRRQQLSAELGLNEAQIKIWFQNKRAKIKK
Amphioxus_En_En_ANTP                 KRPRTAFTSEQLQRLKKEFQE-NRYLTEQRRQDLARELKLNESQIKIWFQNKRAKIKK
ANTP_NKL_Clade_Hmx_Cgi_Hmx1          KKTRTVFSRHQIFYLESAFDA-KRYLSSTERSEIASTLNLTETQVKVWFQNRRNKWKS
ANTP_NKL_Clade_Hmx_Pfuc_3906_1       KKTRTVFSRHQIFYLESAFDT-KRYLSSTERAEIASALNLTETQVKVWFQNRRNKWKS
Beetle_Hmx_Nk5_Hmx_ANTP              KKTRTVFSRSQVFQLESTFDM-KRYLSSSERAGLAASLHLTETQVKIWFQNRRNKWKR
Amphioxus_Hmx_Nk5_Hmx_ANTP           KKTRTVFSRQQVFQLESTFDM-KRYLSSSERAGLAASLHLTETQVKIWFQNRRNKWKR
ANTP_NKL_Clade_I_Cgi_NKL             KKARTAFSTEQIHDLEKRYQA-QKYLPANERQALAEKLGLSDQQVKTWFQNRRMKEKR
ANTP_NKL_Clade_I_Ctel_1523787_       KKARTTFTADQTIILEETYVH-RRYLPAAERRRLAARLRLGDQQVKTWFQNRRMKEKR
ANTP_NKL_Clade_I_Ctel_1525632_       KKPRTTFLPHQLRCLEETYAK-RRYVSAAEREKIAIMLQLTDVQVKTWFQNRRMKARR
ANTP_NKL_Clade_I_Lgig_1410826_       KKARTAFSNDQIQKLEKRFRE-QKYLAASERSELADKLHLSDQQVKTWFQNRRMKEKR
ANTP_NKL_Clade_I_Pfuc_9305_1_6       KKARTAFTTEQINELEKRYQA-QKYLPANERSILAERLGLADQQVKTWFQNRRMKEKR
Beetle_abd_A_Hox6_8_ANTP             RRGRQTYTRFQTLELEKEFHF-NHYLTRRRRIEIAHALCLTERQIKIWFQNRRMKLKK
Beetle_Abd_B_Hox9_13_15__ANTP        RKKRKPYSKFQTLELEKEFLF-NAYVSKQKRWELARNLNLTERQVKIWFQNRRMKNKK
Beetle_Abox_Abox_ANTP                RKPRQAYSAKQLERLESEFKV-DKYLSVSKRMELSKALNLTEVQIKTWFQNRRTKWKK
Beetle_Bap_Nk3_ANTP                  KRSRAAFTHAQVFELERRFSQ-QRYLSGPERADLAQALKLTETQVKIWYQNRRYKTKR
Beetle_Bari_Bari_ANTP                RRRRTAFTHAQLAYLERKFRC-QKYLSVADRSDVADALNLSETQVKTWYQNRRTKWKR
Beetle_Bsh_Bsx_ANTP                  RKARTVFSDPQLTGLEKRFSS-QRYLSTPERVELASALSLSETQVKTWFQNRRMKHKK
Beetle_Btn_Meox_ANTP                 RKERTAFTKEQIKQLENEFAH-SNYLTRLRRYEIAVALDLTERQVKVWFQNRRMKWKR
Beetle_B_H_Barhl_ANTP                RKARTAFTDLQLQTLEKSFER-QKYLSVQDRMELAAKLSLTDTQVKTWYQNRRIEWTL
Beetle_C15_Tlx_ANTP                  KKPRTSFTRLQIAELEKRFHK-QKYLASAERAALAKTLKMTDAQVKTWFQNRRTKWRR
Beetle_Cad1_Cdx_ANTP                 DKYRVVYTDLQRIELEKEFTFVSKYITIKRKSELAENLGLSERQIKIWFQNRRAKERK
Beetle_Cad2_Cdx_ANTP                 DKYRVVYTDHQRVELEKEFYY-SRYITIRRKAELANSLGLSERQVKIWFQNRRAKERK
Beetle_Cx_Hox5_ANTP                  KRQRTSYTRYQTLELEKEFHF-NRYLTRRRRIEIAHALCLTERQIKIWFQNRRMKWKK
Beetle_Dbx_Dbx_ANTP                  MMRRAVFSDLQRKGLERRFQI-QKYISKPDRKKLAEKLGLKDSQVKIWFQNRRMKWRN
Beetle_Dfd_Hox4_ANTP                 KRQRTAYTRHQILELEKEFHY-NRYLTRRRRIEIAHTLVLSERQIKIWFQNRRMKWKK
Beetle_Dll_Dlx_ANTP                  RKPRTIYSSLQLQQLNRRFQR-TQYLALPERAELAASLGLTQTQVKIWFQNRRSKYKK
Beetle_Dr1_Msx_ANTP                  RKPRTPFTTQQLLALEKKFRD-KQYLSIAERAEFSSSLRLTETQVKIWFQNRRAKAKR
Beetle_Dr2_Msx_ANTP                  RKPRTPFTTQQLLALEKKFRD-KQYLSIAERAEFSSSLRLTETQVKIWFQNRRAKAKR
Beetle_Ems_Emx_ANTP                  KRIRTAFSPSQLLKLEHAFEK-NHYVVGAERKQLAQSLSLTETQVKVWFQNRRTKHKR
Beetle_Eve_Evx_ANTP                  RRYRTAFTREQLSRLEKEFHR-ENYVSRPRRCELAAQLHLPESTIKVWFQNRRMKDKR
Beetle_Exex_Mnx_ANTP                 RRPRTAFTSQQLLELEKQFRQ-NKYLSRPKRFEVATSLMLTETQVKIWFQNRRMKWKR
Beetle_ftz_Hox6_8_ANTP               KRTRQTYTRYQTLELEKEFHF-NKYLTRRRRIEIAESLRLTERQIKIWFQNRRMKAKK
Beetle_H2_0_Hlx_ANTP                 SWSRAVFSNLQRKGLERRFQL-QKYITKPDRRQLAATLGLTDAQVKVWFQNRRMKWRH
Beetle_Hex_Hhex_ANTP                 KGGQVRFTANQTDALEKRFTS-HKYLSPEDRKLLAESLKLTDRQVKTWFQNRRAKWRR
Beetle_Hgtx_Nk6_ANTP                 KHTRPTFSGQQIFALEKTFEQ-TKYLAGPERAKLAYALGMTESQVKVWFQNRRTKWRK
Beetle_Ind_Gsx_ANTP                  KRIRTAFTSTQLLELEREFAS-NMYLSRLRRIEIATCLRLSEKQVKIWFQNRRVKYKK
Beetle_lab_Hox1_ANTP                 NTGRTNFTNKQLTELEKEFHF-NKYLTRARRIEIASALQLNETQVKIWFQNRRMKQKK
Beetle_Lbx_Lbx_ANTP                  RKSRTAFTNHQIFELEKRFLY-QKYLSPADRDEIASSLGLTNAQVITWFQNRRAKLKR
Beetle_Msxlx_Msxlx_ANTP              RHPRIPFTSYQLSVLEEKFKQ-SPYLSSEEVTMLSRQLQLVDVRVKIWFQNRRARERR
Beetle_mxp_Hox2_ANTP                 RRLRTAYTNTQLLELEKEFHF-NKYLCRPRRIEIAASLDLTERQVKVWFQNRRMKHKR
Beetle_Nedx_Nedx_ANTP                KRPRTAFTASQIKSLEAEFER-NKYLSVAKRCQLSKTLKLTETQIKIWFQNRRTKWKR
Beetle_NK7_Nk7_ANTP                  KKARTTFTGRQIFELEKQFEM-KKYLSSSERSEMAKLLNVTETQVKIWFQNRRTKWKK
Beetle_Not_Noto_ANTP                 KRVRTIFTPEQLERLEAEFER-QQYMVGPERLYLAHTLQLTEAQVKVWFQNRRIKWRK
Beetle_Ro_Ro_ANTP                    RRQRTTFSSEQTLRLEIEYQR-SEYISRGRRCELAEALKLSETQIKIWFQNRRAKDKR
Beetle_Scro_Nk2_1_ANTP               RKRRVLFTQAQVYELERRFKQ-QKYLSAPEREHLASLIHLTPTQVKIWFQNHRYKCKR
Beetle_Slou_Nk1_ANTP                 RRARTAFTYEQLVALENKFKT-TRYLSVCERLNLALSLSLTETQVKIWFQNRRTKWKK
Beetle_Tin_Nk4_ANTP                  RKPRVLFSQAQVYELERRFKQ-QKYLSAPEREQMAQGLKLTPTQVKIWFQNRRYKSKR
Beetle_Unpg_Gbx_ANTP                 RRRRTAFTSEQLLELEREFHA-KKYLSLTERSQIASALRLSEVQVKIWFQNRRAKWKR
Beetle_Utx_Hox6_8_ANTP               RRGRQTYTRYQTLELEKEFHT-NHYLTRRRRIEMAHALCLTERQIKIWFQNRRMKLKK
```

PRD genes

```
                                         10        20        30        40        50        60
                                ....|....|....|....|....|....|....|....|....|....|....|....|....|..
Beetle_Repo_Repo_PRD            -KKTRTTFTAFQLEELERAFE-RAPYPDVFAREELALKLSLSESRVQV-----WF--QNRRAKWRK-
Beetle_Otp_Otp_PRD              -KRHRTRFTPAQLNELERCFS-KTHYPDIFMREEIAMRIGLTESRVQV-----WF--QNRRAKWKK-
Beetle_Ey_Pax4_6_PRD            -QRNRTSFTNDQIDSLEKEFE-RTHYPDVFARERLAAKIGLPEARIQV-----WF--SNRRAKWRR-
Beetle_Toy_Pax4_6_PRD           -QRNRTSFTNEQIDSLEKEFE-RTHYPDVFARERLAEKIGLPEARIQV-----WF--SNRRAKWRR-
Beetle_Oc1_Otx_PRD              -RRERTTFTRAQLDLLEGLFA-KTRYPDIFMREEVAVKINLPESRVQV-----WF--KNRRAKCRQ-
Beetle_Oc2_Otx_PRD              -RRERTTFTRAQLDVLEALFG-KTRYPDIFMREEVALKINLPESRVQV-----WF--KNRRAKCRQ-
Beetle_Gsc_Gsc_PRD              -RRHRTIFSEEQLEQLEATFE-QTHYPDVVLREQLALKVDLKEERVEV-----WF--KNRRAKWRK-
Beetle_Vsx_Vsx_PRD              -RRHRTIFTSYQLEELEKAFK-DAHYPDVYAREMLSLKTDLPEDRIQV-----WF--QNRRAKWRK-
Beetle_Prop_Prop_PRD            -RRHRTTFTQEQLAELEAAFA-KSHYPDIYCREELARSTKLNEARIQV-----WF--QNRRAKYRK-
Beetle_Phox_Phox_PRD            -RRIRTTFTSAQLKELERAFQ-ETHYPDIYTREEIAMKIDLTEARVQV-----WF--QNRRAKFRK-
Beetle_Eyg_Pax4_6_PRD           -RRNRTTFSPDQLEELEKEFE-KSHYPCVSTRERLASKTSLSEARVQV-----WF--SNRRAKWRR-
Beetle_CG11294_CG11294_PRD      -RRNRTTFSPQQLQELEALFQ-KTHYPDVFLREEVALRISLSEARVQV-----WF--QNRRAKWRK-
Beetle_Drgx_Drgx_PRD            -RRNRTTFTLQQLEELESAFA-QTHYPDVFTREDLAMKINLTEARVQV-----WF--QNRRAKWRK-
Beetle_Prrx_Prrx_PRD            -RRNRTTFTTVQLAALEKVFE-KTHYPDAFVREDLAAKVNLSEARVQV-----WF--QNRRAKFRR-
Beetle_Rx_Rax_PRD               -RRNRTTFTTYQLHELERAFE-KSHYPDVYSREELAMKVNLPEVRVQV-----WF--QNRRAKWRR-
Beetle_Ptx1_Pitx_PRD            -RRQRTHFTSQQLQELEATFA-RNRYPDMSTREEIAMWTNLTEARVRV-----WF--KNRRAKWRK-
Beetle_Unc4_Uncx_PRD            -RRSRTNFNSWQLEELERAFL-ASHYPDVFMREALAMRLDLKESRVAV-----WF--QNRRAKWRK-
Beetle_Shox_Shox_PRD            -RRSRTNFTLEQLNELERLFD-ETHYPDAFMREELSQRLGLSEARVQV-----WF--QNRRAKCRK-
Beetle_Gsbn_Pax3_7_PRD          -RRSRTTFSGEQLEALERAFS-RTQYPDVYTREELAQQTGLTEARIQV-----WF--SNRRARLRK-
Beetle_Prd_Pax3_7_PRD           -RRSRTTFTAHQLDELEKAFE-RTQYPDIYTREELAQRTKLTEARIQV-----WF--SNRRARLRK-
Beetle_Gsb_Pax3_7_PRD           -RRSRTTFTGEQLEALERAFG-RTQYPDVYTREELAQKTKLTEARVQV-----WF--SNRRARLRK-
Amphioxus_Otp_Otp_PRD           -KRHRTRFTPAQLQELERSFS-KTHYPDIFMREELALRIGLTESRVQV-----WF--QNRRAKWKK-
Amphioxus_UncxC_Uncx_PRD        -MRVRTNFTSWQLEALERAFE-TTHYPDIFMREALALRLDLMESRVQV-----WF--QNRRAKWRK-
Amphioxus_Pax6_Pax4_6_PRD       -QRNRTSFTQEQIEALEKEFE-RTHYPDVFARERLAAKIDLPEARIQV-----WF--SNRRAKWRR-
Amphioxus_Otx_Otx_PRD           -RRERTTFTRAQLDVLEALFA-KTRYPDIFMREEVALKINLPESRVQV-----WF--KNRRAKCRQ-
Amphioxus_Gsc_Gsc_PRD           -RRHRTIFTEEQLELLEKTFE-KTHYPDVLLREELAMKVELKEERVEV-----WF--KNRRAKWRK-
Amphioxus_Vsx_Vsx_PRD           -RRHRTIFTSYQLEELEKAFN-EAHYPDVYAREMLAMKTDLPEDRIQV-----WF--QNRRAKWRK-
Amphioxus_Prop_Prop_PRD         -RRHRTTFTQEQLAQLEEAFS-KSHYPDIYVREELARATKLNEARIQV-----WF--QNRRAKYRK-
Amphioxus_Phox_Phox_PRD         -RRIRTTFTSAQLKELERVFA-ETHYPDIYTREELALKIDLTEARVQV-----WF--QNRRAKFRK-
Amphioxus_Drgx_Drgx_PRD         -RRNRTTFTLHQLEQLEAVFA-QTHYPDVFTREELAMKINLTEARVQV-----WF--QNRRAKWRK-
Amphioxus_Alx_Alx_PRD           -RRNRTTFTSFQLEEMERVFQ-KTHYPDVYMREQLALRADLTEARVQV-----WF--QNRRAKWRK-
PRD_Clade_I_Cgi_PRD6            SKRNRTTFSTKQLQELEKVFR-KTHYPDVFLREKLASKIKLPESRIQV-----WF--QNRRAKWRKR
PRD_Clade_I_Ctel_1513785_90_8   QRRNRTTFSAEQLRELEAVFT-HTHYPDCTLREQIADKVGLTEARVQV-----WF--QNRRAKWRKQ
PRD_Clade_I_Pfuc_5_1_65164      GKRNRTTFSTKQLQELERAFR-KTHYPDVFLREKLALKIKLPESRIQL-----YD--FPSYLEYRIL
PRD_Clade_II_Cgi_PRD8           QRRNRTTFTSDQLRELEAVFQ-HTHYPDCTLREQIAEKVDLTEARVQV-----WF--QNRRAKWRKQ
PRD_Clade_II_Ctel_1512825_85_7  VKRNRTTFSTKQLHELERAFR-KTHYPDIFMREKLAMRINLPESRIQV-----WF--QNRRAKWRKR
PRD_Clade_II_Lgig_1413675_88_4  QRRNRTTFSADQLRELETVFQ-HTHYPDCTLREHIADKVDLTEARVQV-----WF--QNRRAKWRKQ
PRD_Clade_III_Beetle_CG11294_C  QRRNRTTFSPQQLQELEALFQ-KTHYPDVFLREEVALRISLSEARVQV-----WF--QNRRAKWRKQ
PRD_Clade_III_Ctel_1502880_82_  QRRNRTTFTPQQLASLEELFS-RTHYPDIFVREELASRITLTEARVQV-----WF--QNRRAKWRKN
PRD_Clade_III_Lgig_1413475_78_  QKRQRTSFMPSQVMILEETFL-HTQYPDIFLREELSQKISLCEARIQV-----WF--QNRRARWRR-
PRD_Clade_IV_Cgi_PRD1           KRRFRTTFTADQLRCLENVFR-LTHYPDVNSREELSQKTGLPEARVQI-----WF--QNRRAKWRKY
PRD_Clade_IV_Ctel_1516887_86_0  PRRSRTTFTSEQISDLELTFK-LTHYPDVSSREKLAIKTGLSETRIQI-----WF--QNRRAKWRKN
PRD_Clade_IV_Lgig_1419487_81_3  QRRFRTTFTTEQLHDLENVFL-ITHYPDVNTRDELSIKTGLSEERVQI-----WF--QNRRAKWRKY
PRD_Clade_IV_Pfuc_130248_1_717  KRRFRTTFTAGQLKDLEGVFR-MTHYPDVNMREELSRKTGLPEARVQI-----WF--QNRRAKWRKY
PRD_Clade_IV_Pfuc_96805_1_2806  KRRFRTTFTAGQLKDLEGVFR-MTHYPDVNMREELSRKTGLPEARVQI-----WF--QNRRAKWRKY
PRD_Clade_V_Cgi_PRD4            GTKPRTKYSPEQLELLEGAFL-DSQYPDTETLEDLAERLDVSTDKVSV-----WF--QNRRSKFKRQ
PRD_Clade_V_Cgi_PRD5            PTRSRIKYRQDQLETLEESFR-SHQYPDSESIENLAEKVGVSSERVAI-----WF--QNRRAKFKRE
PRD_Clade_V_Ctel_1503273_58_1   IRRARTNFSPEQLSAMEIVFE-RTQYPDVKLLSTLSHKLQLPIEKLCT-----WF--QNRRSKFRRA
PRD_Clade_V_Ctel_1522777_61_6   IRRVRTNFSQEQLSAMEVIFE-HNQYPDVKILSTLSDKLQLPVEKLCT-----WF--QNRRSKYRRL
PRD_Clade_V_EmW_000533200       -GRRRVRYEDWQIEILTREFE-RNHRPSSSVYARIADATNVSTKNVRI-----WF--KNRRANG---
PRD_Clade_V_Lgig_1416133_59_6   RRRERTKYSRKQLNILERIFQ-ENHYPDSYVFDQLCDTLNLDIEKLAT-----WF--QNRRCKEKRL
PRD_Clade_V_Lgig_1416135_43_7   RRRERTKYS---LEYFRRIFQ-ENHYPDSYVFDQLCDTLNLDIEKLAT-----WF--QNRRCKEKRL
PRD_Clade_V_Lgig_1427767_62_7   QPRNRTKYTAAQLNALESLFM-VSQYPDSLCLEDLCKSLGVTVERIQI-----WF--QNRRSKFKRQ
PRD_Clade_V_Lgig_1427769_66_5   QPRNRTKYTAAQLNTMESLFL-VSQYPDSQALEELAKSLAVTVERISI-----WF--QNRRSKFKRQ
PRD_Clade_V_Pfuc_1245_1_65923   ETRYRTKYSDEQLELMEKTFA-KNQYPDSEVLADLSDRLDIAHDKLIKRVCTKLFRFRTTRSEQPRL
PRD_Clade_VI_Ctel_1510374_58_8  QAKQRITFKRHEIECLDRIFL-EVEYPEADLIDRLAQELSLPTNNVKV-----WF--QNKRSRQRKR
PRD_Clade_VI_Lgig_1408680_70_5  KKNSRTNYSPQQVRSLEKIFL-DTPYPESEVMEQLSRDLDIPEKNLKI-----WF--QNKRARWRKR
PRD_Clade_VI_Lgig_1408686_70_6  KKNSRTNYSPQQVRSLEKIFL-DTPYPESEVMEQLSRDLDIPEKNLKI-----WF--QNKRARWRKR
PRD_Clade_VI_Lgig_1408687_70_5  KKNSRTNYSPQQVRSLEKIFL-DTPYPESEVMEQLSRDLDIPEKNLKI-----WF--QNKRARWRKR
PRD_Clade_VI_Lgig_1413135_53_9  KRKGRISYTTKQLNAMDRAFE-EDPYPKPDLLDHLSDELQIPVSKLKV-----WF--QNKRARARKR
PRD_Clade_VI_Lgig_1413416_76_3  KKNTRTNYSPQQVRSLEKIFL-DTPYPEPEVMEQLSRDLDIPEKNLKI-----WF--QNKRARWRKR
PRD_Clade_VI_Lgig_1416844_60_8  AKRIRSYFSPTQTKKMEDVFV-VDPFPDKDQIKQLSTSLDISERSLQV-----WF--QNRRARQRKA
PRD_Clade_VI_Lgig_1420675_61_5  SKRHRTHFTSIQVEKLEEIFT-ANQNPYRDLIVHLSTSLNLTHKNILI-----WF--KNRRAKEKRQ
PRD_Clade_VI_Lgig_1423102_62_5  RKRHRTHFTPQQVEKLEEIFTQQTSSPYRELILQLSTTLNLTDKNIAI-----WF--KNRRAKERKQ
PRD_Clade_VI_Lgig_1423104_62_5  RKRHRTHFTPQQVEKLEEIFTQQTSSPYRELILQLSTTLNLTDKNIAI-----WF--KNRRAKERKQ
PRD_Clade_VI_Lgig_1428434_53_3  KKRNRVHYSADQIRVFERLFV-KNNFPDRETLESICEDMDISHKNAQV-----WF--QNRRARSRRD
PRD_Clade_VI_Lgig_1428515_76_2  KKNSRTNYSPQQVRSLEKIFL-DTPYPESEVMEQLSRDLDIPEKNLKI-----WF--QNKRARWRK-
PRD_Clade_VI_Lgig_1428521_76_2  KKNSRTNYSPQQVRSLEKIFL-DTPYPESEVMEQLSRDLDIPEKNLKI-----WF--QNKRARWRK-
PRD_Clade_VI_Lgig_1428776_58_1  KKRSRQTFTAYQTQTLEAHFH-RNNFPDKSAIDKICMSMDINETRLQN-----WF--KNRRARSRRD
PRD_Clade_VI_Lgig_1430968_78_8  -KKARTNYTPEQVRGLEKVFL-ETPYPEPEAMEKLSKELEIPEQKLKV-----WF--QNKRARWRR-
PRD_Clade_VI_Lgig_1431008_76_3  KKNTRTNYSPQQVRSLEKIFL-DTPYPEPEVMEQLSRDLDIPEKNLKI-----WF--QNKRARWRKR
PRD_Clade_VI_Pfuc_2703_1_01206  ----------------------------------MTKMRDVGHHDVTV-----WF--KNRRAKFRKK
PRD_Clade_VI_Pfuc_64133_1_6363  RKKARTNYSPSQVQALEKVFH-ENPYPDSEKMEALSLELDIPEGKIK--------------------
PRD_Clade_VI_Pfuc_729_1_22278   RKKTRTNYSPSQVQALEKVFH-ENPYPDSEKMEALSLELDIPEGKIKL-----FK--TSRFAWVPSQ
```

LIM genes

```
                                     10        20        30        40        50
                                ....|....|....|....|....|....|....|....|....|....|....|..
Amphioxus_Lhx2_9_a_Lhx2_9_LIM   KRIRTSFKHHQLRTLKSYFAINHNPDSKDLQQLAQKTGLTKRVLQVWFQNARAKHRR
Amphioxus_Lhx2_9_b_Lhx2_9_LIM   KRMRTSFKHHQLRAMKSYFALNHNPDAKDLKQLAQKTGLTKRVLQVWFQNARAKYRR
Amphioxus_Lmx_Lmx_LIM           KRPRTILTTQQRREFKALFEVSPKPCRKVRETLAAETGLSVRVVQVWFQNQRAKMKK
Amphioxus_Lhx3_4_Lhx3_4_LIM     KRPRTTITAKQLETLKQAYQNSPKPARHVREQLSQETGLDMRVVQVWFQNRRAKEKR
Amphioxus_Lhx6_8_Lhx6_8_LIM     KRVRTTFTEEQLRVLQANFNIDSNPDGQDLERIAQITGLSKRVTQVWFQNSRARQKK
Amphioxus_Lhx1_5_Lhx1_5_LIM     RGPRTTIKAKQLETLKAAFAATPKPTRHIREQLAQETGLNMRVIQVWFQNRRSKERR
Amphioxus_Isl_Isl_LIM           TRVRTVLNEKQLHTLRTCYAANPRPDALMKEQLVEMTGLSPRVIRVWFQNKRCKDKK
Beetle_Ap1_Lhx2_9_LIM           KRMRTSFKHHQLRTMKSYFAINHNPDAKDLKQLSQKTGLPKRVLQVWFQNARAKWRR
Beetle_Ap2_Lhx2_9_LIM           KRMRTSFKHHQLRTMKTYFAINQNPDAKDLKQLAQKTGLSKRVLQVWFQNARAKWRR
Beetle_Lmxb_Lmx_LIM             KRPRTILTSAQRRQFKASFEVSPKPCRKVREALAKETGLSVRVVQVWFQNQRAKMKK
Beetle_Lmxa_Lmx_LIM             KRPRTILTTQQRRAFKASFEVSPKPCRKVREALAKDTGLSVRIVQVWFQNQRAKMKK
Beetle_Lim3_Lhx3_4_LIM          KRPRTTITAKQLETLKNAYNNSPKPARHVREQLSQDTGLDMRVVQVWFQNRRAKEKR
Beetle_Awh_Lhx6_8_LIM           KRVRTTFTEEQLQVLQANFQLDSNPDGQDLERIAQITGLSKRVTQVWFQNSRARQKK
Beetle_Lim1_Lhx1_5_LIM          RGPRTTIKAKQLEILKTAFSQTPKPTRHIREQLAKETGLPMRVIQVWFQNKRSKERR
Beetle_Tup_Isl_LIM              TRVRTVLNEKQLHTLRTCYAANPRPDALMKEQLVEMTGLSPRVIRVWFQNKRCKDKK
LIM_Clade_I_Clonorchis_sinensi  KRMRTSLTDDQRLHMQRVYESNPRPSKEAREKLANELGVPLRVVQVWFQNQRARDRR
LIM_Clade_I_Smp_163600          RRIRTSLTDEQRYRLQEAYELNIRPSKSIREALASELGVPMHVVQVWFQNQRARDKR
```

SIX genes

```
                                  10        20        30        40        50
                             ....|....|....|....|....|....|....|....|....|....|....|..
Amphioxus_Six1_2_Six1_2_SINE EETSYCFKEKSRGVLREWYAHNPYPSPREKRELAEATGLTTTQVSNWFKNRRQRDRA
Amphioxus_Six3_6_Six3_6_SINE EQKTHCFKERTRSLLREWYLQDPYPNPQKKRELAQATGLTPTQVGNWFKNRRQRDRA
Amphioxus_Six4_5_Six4_5_SINE EETVYCFKEKARQALKEMYNNNRYPTPDEKRNLAKKTGLTLTQVSNWFKNRRQRDRT
Beetle_So_Six1_2_SINE        EETSYCFKEKSRSVLRDWYSHNPYPSPREKRELADATGLTTTQVSNWFKNRRQRDRA
Beetle_Optix_Six3_6_SINE     EQKTHCFKERTRSLLREWYLQDPYPNPTKKRELAQATGLTPTQVGNWFKNRRQRDRA
Beetle_Six4_Six4_5_SINE      EETVYCFKERSRNALKECYARNRYPTPDEKRALAKRTGLTLTQVSNWFKNRRQRDRT
SIX_Clade_I_EmW_000130200    ---SYYFKEKSRRLLRQQYAENPYPSQWEKKELARRTGLTAVQVSNWFKNRRQRDRT
SIX_Clade_I_Smp_039470       ----YYFKQNIRYLLINYYQYNKYPNSIEKYEISRKTGLTLTQVSNWFKNHRQRDKS
SIX_Clade_I_Smp_127240       -QVTYYFKDKSRNYLAEQFVHNSYPSIVEKKFMAKKSGLTITQVSNWFKNRRQRDKT
```

CUT genes

```
                                     10        20        30        40        50
                                ....|....|....|....|....|....|....|....|....|....|....|..
Beetle_Ct_Cux_CUT               KKQRVLFSEEQKEALRLAFALDPYPNVATIEFLASELGLSSRTITNWFHNHRMRLKQ
Beetle_Onecut_Onecut_CUT        KKPRLVFTDLQRRTLQAIFKETKRPSKEMQVTIARQLGLEPTTVGNFFMNARRRSMD
Amphioxus_Onecut_Onecut_CUT     KKPRLVFTDLQRRTLHAIFKENKRPSKEMQAQIAKQLGLDLSTVCNFFMNARRRSQD
Amphioxus_Cux_Cux_CUT           KKQRVVLSPEEKEALRKAYEQEPYPSPSTIEYLAAKLNLRPCTVTNWFHNYRSRLRR
Cgi_Cux                         KRPRVFFTEEQKDKLRMAYNQDPYPNQNTIEALANELNVGVKTVINWFHNHRMRAKQ
CUT_Clade_I_Ctel_1526154_73_1   RRARTMFSESATSFLEAEFACNPYPNVETREELAVRLEVQEARVHTWFQNKRSRAKR
CUT_Clade_I_Lgig_1430162_73_1   RRGRTAFSENAIDRLEGVFMVNRYPDINLRETLAQQLGVAESRIQVWFQNRRSRGRR
CUT_Clade_I_Lgig_1413319_72_2   RRGRTAFSETAITRLEDVFMVNRYPDITLRESLALQLGVAESRIQVWFQNRRSRGRR
CUT_Clade_I_Lgig_1413498_72_5   RRGRTAFSETAITRLEDVFMVNRYPDITLRETLALQLGVAESRIQVWFQNRRSRGRR
CUT_Clade_I_Lgig_1430159_74_6   RRNRTYFSDESLDRLETVFNENPYPDIVQREALGLECGVTEARIQVWFQNRRSRNRR
CUT_Clade_I_EmW_000559800       RRPRTRLSPAQISMLVQAFEISPSPDYATRVQLALSTERP---------GRKAALEA
CUT_Clade_I_EmW_000280300       RRPRTRLSPAQISMLVQAFEISPSPDYATRVQLALSTGLPEDTVHIWFQNRKARQKG
CUT_Clade_I_EmW_000280500       RRPRTRLSPAQISMLVQAFEISPSPDYATRVQLALSTGLPEDTVHIWFQNRKARQKG
CUT_Clade_I_Pfuc_4520_1_30706-  RRTRTVFSDDALDALEDAFQENCYPDVFQRYSLAQDIGEEESRIQVWFQNRRARSKR
CUT_Clade_I_Cgi_CUT1            SGRRGKFTLFQLMVLCRFFEEDQNPSLNTRMLLAEKLLVSLERITIWFQNQRARGFP
CUT_Clade_I_Cgi_CUT2            SGRYSTFSMFALDILKEFYHINGFPSKSERTKICEIIGETEKRVQNWFKGQRKVDRR
CUT_Clade_I_Pfuc_12964_1_53883  SRTRSSYSYATLDIFQSVFAINGYPSRQERNRLAELTGETHLRVASWFKYQRARAHK
CUT_Clade_I_EmW_000273500       -------------MLVQAFEISPSPDYATRVQLALSTGLPEDTVHIWFQKRKARQKD
CUT_Clade_I_Ctel_1516427_52_3   -----LCARLKLRRLESAYLDDSYPDVNDRTRLALDLGVNEDRIQVWFQNRRARGRR
```

TALE genes

```
                                        10        20        30        40        50        60
                                ....|....|....|....|....|....|....|....|....|....|....|....|
Beetle_Ara_Irx_TALE             AARRKNATRESTATLKAWLNEHKKNPYPTKGEKIMLAIITKMTLTQVSTWFANARRRLKK
Beetle_Mirr_Irx_TALE            GARRKNATRETTSTLKAWLNEHKKNPYPTKGEKIMLAIITKMTLTQVSTWFANARRRLKK
Beetle_Tgif2_Tgif_TALE          IKRRGHLPKDAVKILKNWLYEHRFNAYPTEVEKNILSQETNLTVLQISNWFINARRRYLP
Beetle_Pknox_Pknox_TALE         KQKRGVLPKHATSVMRSWLFQHLVHPYPTEDEKRHIAAQTNLTLLQVNNWFINARRRILQ
Beetle_Hth_Meis_TALE            QKKRGIFPKVATNILRAWLFQHLTHPYPSEDQKKQLAQDTGLTILQVNNWFINARRRIVQ
Beetle_Tgif1_Tgif_TALE          RKRRGNLPKHSVKILKRWLYEHRYNAYPSDAEKMTLSQEANLTVLQVCNWFINARRRILP
Beetle_Mkx_Mkx_TALE             RPPKRLFTPEIKRFLKDWLVRRRENPYPNRDEKKNLALQTGLTYIQVCNWFANWRRKLKN
Beetle_Exd_Pbx_TALE             RRKRRNFSKQASEILNEYFYSHLSNPYPSEEAKEELARKCGITVSQVSNWFGNKRIRYKK
Amphioxus_IrxA_Irx_TALE         GARRKNATRETTATLKAWLMEHRKNPYPTKGEKIMLAIITKMTLTQVSTWFANARRRLKK
Amphioxus_IrxB2_Irx_TALE        GTPRKNATRDATSTLKAWLNEHKKNPYPTKGEKIMLAIITKMTLTQVSTWFANARRRLKK
Amphioxus_IrxB1_Irx_TALE        GTRRKNATRDATSTLKAWLNEHRKNPYPTKGEKIMLAIITKMTLTQVSTWFANARRRLKK
Amphioxus_Pknox_Pknox_TALE      NGKRGILPKQATDVMRSWLFQHIVHPYPTEDEKRAIANQTNLTLLQVNNWFINARRRILQ
Amphioxus_Meis_Meis_TALE        QKKRGIFPKVATNIMRAWLFQHLTHPYPSEEQKKQLAQDTGLTILQVNNWFINARRRIVQ
Amphioxus_Tgif_Tgif_TALE        RKRRGNLPKEAVQILRAWLYDHRYNAYPTDAEKLDLAREAGLTVLQVCNWFINARRRILP
Amphioxus_Pbx_Pbx_TALE          RRKRRNFSKQATEVLNEYFYSHLSNPYPSEEAKEELARKCGITVSQVSNWFGNKRIRFKK
Amphioxus_Mkx_Mkx_TALE          VRHKRQVMQDMARPLKQWLIKHRDNPYPTKTEKILLALTSQMTLVQVSNWFANARRRLKN
TALE_Clade_I_Cgi_TALE2          SKKHPSLPKEAVAIMLEWLRQHKDNPYPNDDEKAMLIKQTGLTINQINYWFTNARRRILP
TALE_Clade_I_Ctel_1513294_24_8  -----------------WLRKHQDNPYPNDDEKEMLIQKTKLTINQINYWFTNARRRILP
TALE_Clade_I_Lgig_1414665_30_1  -----------------WLRNHKDNPYPNDDEKAMLIKQTGLTINQINYWFTNARRRILP
TALE_Clade_I_Pfuc_24948_1_1165  ----------AVAIMLDWLRQHKDNPYPNDDEKAMLIKQTGLTINQINYWFTNARRRILP
TALE_Clade_II_Cgi_TALE1         RPRSRQLNSKATSIMSHWFEKNIDHPYPSDEQKEQLAREGGITVAQVKAWFANKRNRTSN
TALE_Clade_II_Pfuc_13151_1_322  KPRSRSLNAKATALMMEWFEKHIENPYPSDAEKEELARAGGISVPQVKAWFANRRNRTSN
TALE_Clade_II_Pfuc_13478_1_323  KPRSRSLNAKATALMMEWFEKHIENPYPSDAEKEELARAGGISVPQVKAWFANRRNRTSN
TALE_Clade_III_Cgi_TALE3        QRHNQPLNLKAVRIMTEWYDRHEENPYPSKSEKEIMAKEGGISVTQVKSWFANKRNRSNN
TALE_Clade_III_EmW_000753000    --PNSQLNPNAVAIMDEWYRAHLDRPYPNKEEKLRMAIAGDITETQVGSWFANRRNRSNN
TALE_Clade_III_EmW_001016600    ----KLLSTRATQILDGWYETNTEWPYPSKAEKQMMASAGGITIEQVNSWFANRRNRSQN
TALE_Clade_III_Pfuc_98062_1_56  QRHNQPLNLKAVSIMTGWYESHLENPYPTKAEKEEMARQGGITLTQVKSWFANKRNRSNN
TALE_Clade_III_Smp_063520       -TRNRPLNQTALSVMESWYTNHVDNPYPTTAEKEELAALGGITVIQVSSWFANRRTRTA-
TALE_Clade_IV_Cgi_TALE14        SSNYKQLHPQARRVLGEWYDVHMNNPYPSDEEKSQLAERAGITEQQVKSWFANKRSRANN
TALE_Clade_IV_Clonorchis_sinen  RPHRQNFTPTQNRILTEWYQTHQSKPYPSTDDTKELAIISGLSYSQVKKWFANKRARSSS
TALE_Clade_IV_Ctel_1499505_38_  ----GIMTPDAVSILLRWYEQHQDHPYPSNDTAVLLAQTAKLSVRQVKKWFANRRRR---
TALE_Clade_IV_Ctel_1505080_24_  -------PAEAVVLMSKWYQENFHYPYPDKEDMQHFASAGGITVGQVKKWMANKRVR---
TALE_Clade_IV_Ctel_1526117_32_  --RRRFLSPESVRILSDWYTEHEDHPYPSDQIVERLANRANISVFQVKKWMANKRVR---
TALE_Clade_IV_EmW_000427000     RPHRQNFTALQNRILTDWYNSHHYKPYPSTEDTKMLAQKSELTYSQVKKWFANKRARTS-
TALE_Clade_IV_Pfuc_1892_1_6613  TTAFQQISQEAKQILNEWYDTHISHPYPSDEERSMLATQTGVPESKIKSWFANKRSRSHN
TALE_Clade_IV_Smp_157830        RPHRQNFTPTQNRILTEWYQLHQAKPYPSTDDTKELANISGLSYSQVKKWFANKRARST-
TALE_Clade_V_Cgi_TALE6          ARKTRLLPKRSVKILDGWFTDNISNPYPSRDQTIRLALDCGLTVEQVRKWFANKRNRSRN
TALE_Clade_V_Cgi_TALE7          IPAFQPMAPESRDFLNQWYKDNITHPYPTDQQREELAQQTGLSIQQVKRWLANKRSRANN
TALE_Clade_V_Cgi_TALE8          IPAFQPMAPESRDILNQWYKDNITHPYPTDQQREELAQQTGLSLQQVKRWLANKRSRANN
TALE_Clade_V_Pfuc_255_1_07443   IRKTRLLPKRSVKMLESWFQENISNPYPSRDQTVRLALDCGLTVEQVRKWFANKRNRSRN
TALE_Clade_VI_Cgi_TALE10        IKPRPLLSKRAVQLMEKWYDNHLEHPYPNIDTIEQLATTGNITPEQVKKWFANKRNRSNN
TALE_Clade_VI_Cgi_TALE11        LRSRPSLSQKAVQLMEEWYYSHRDHPYPPHHIIQDLARKGGVREEQVKKWFSNKRNRSRR
TALE_Clade_VI_Cgi_TALE12        IRSRPSLSQRAVRLMEEWYYSHRDHPYPPQHIIQDLARRGGVKEEQVKKWFSNKRNRSRR
TALE_Clade_VI_Cgi_TALE13        LRSRPSLSQKAVQLMEEWYYSHRDNPYPPSHIIQDLARKGGVREEQVKKWFSNKRNRSRR
TALE_Clade_VI_Cgi_TALE9         LKNRTLLTKKAVDMMEEWYLSNLDHPYPCHKVVQSLAVFGNIREEQVRKWFANKRTRQGR
TALE_Clade_VI_Lgig_1410135_44_  TRTRPVLTRNSLKVLEEWYECHLDHPYPTASQVEWLAQVSSLNTEQVKKWFGNKRSRSKN
TALE_Clade_VI_Lgig_1410138_39_  TRTRPVLMRNSLKVLEEWYECHLDHPYPTASQVEWLAQVSSLNTEQVKKWFGNKRSRSKN
TALE_Clade_VI_Lgig_1419427_48_  -RSRPVLSKSTIQKLEAWYTAHEEHPYPNNEVIEDLSNACGITYSQVRKWFANKRNRSK-
TALE_Clade_VI_Pfuc_10095_1_389  LRTRPSLSKQAIRLMEVWYHAHIDHPYPDTQDVETLATAGNITEEQVKKWFANKRNRSRN
TALE_Clade_VI_Pfuc_1442_1_2259  RRARPNLARQAVQLLDDWYRDNMDHPYPGPRTITRLATEGGISEEQVKKWYANKRSRCRN
TALE_Clade_VI_Pfuc_18402_1_400  LRTRPSLSKQAIRLMEVWYHAHIDHPYPDTQDTETLATTGNITEEQVKKWFANKRNRSRN
TALE_Clade_VI_Pfuc_22555_1_403  LRTRPSLSKQAIRLMEVWYHAHIDHPYPNTQDVETLATAGNITEEQVKKWFANKRNRSRN
TALE_Clade_VI_Pfuc_22569_1_621  RRARPNLARKAVQLLDDWYRDNMDHPYPGPRTITRLATEGDISEEQVKKWYANKRSRSRN
TALE_Clade_VI_Pfuc_2547_1_3016  LRTRPSLSQRAIRLMEVWYHAHIDHPYPDTQDVETLATAGNITEEQVKKWFANKRNRSRN
TALE_Clade_VI_Pfuc_312_1_50785  LRTRPSLSQRSIRLMEVWYHAHIDHPYPDTQDVETLATAGNITEEQVKKWFANKRNRSRN
TALE_Clade_VI_Pfuc_6497_1_4544  KPNYQQLNPESRKILNEWYNDHSDHPYPDNEAKQTLAEKAGISEEQVKSWFANKRSRAQN
TALE_Clade_VII_Cgi_TALE4        RKQRASFPKKAILIMEDWYEKNVSHPYPDSITVELIAIQGGITGEQVKKWFGNKRNRSNN
TALE_Clade_VII_Pfuc_6013_1_239  VKPRPLLSKKAVRIMEQWYDDNLEHPYPTPAAYDAIAVEGGIAVEQVKKWFANKRNRSHN
```
