# Supplementary material for: Reinforcing the Egg-Timer: Recruitment of Novel Lophotrochozoa Homeobox Genes to Early and Late Development in the Pacific Oyster
Source: Genome Biol Evol. 2015 Jan 27;7(3):677–88. doi: 10.1093/gbe/evv018 (PMC5322547; doi:10.1093/gbe/evv018)
Supplement: Supplementary Data [file supp_evv018_suppl_data.zip › Supp_Figure_8_PADRE box.pdf]

```
                             10        20        30        40        50        60        70        80        90       100       110       120       130       140
                    ....|....|....|....|....|....|....|....|....|....|....|....|....|....|....|....|....|....|....|....|....|....|....|....|....|....|....|....|
Clade VI  Pfuc_6013 MDV------SASSVDTV------PSPSPCQEPVIDLTKVVAETIFDISSDITSDS--CSDLEDDLSSITDVFDP------------TQCYHDDDVKPTCAYQSPTC--LDR--------------LPEFITSSDVVAMDI
Clade VI  Cgi_TALE4   MDGHQC----------------STSPPVLHLPSVKLHSLKLSDFLDIGFDDSFSEILLPHIEDDLDSLTEVFNPVRDGPSDLLDDTTQCSSNFTTSPESQETSSTV---SSPDVF----NFSQKLVPCAMDFSDISSSPD
Clade VII Cgi_TALE9   MD-------------------------------------------------------------DLDFLETALGP-----------ATPCHLDSSILQDLQQTFKDC--EEPP----------------------ILNIDD
Clade VII Cgi_TALE10  MDLR--VYHYVDDIESDSE---KTSPSPGQPPMIDLSEVIATTIFS---DHSED----SSLTDDLDNLTDVFDP--------------CKTDLDDSGVSQDQVLLC--GDKLRV----------RHRSALSSIEVLEFDT
Clade VII Cgi_TALE11  M----------ASCS--------SSPAVAINFTTTLEDIIKATIFD---------------------------------------------------------------DAP--------------S-------------
Clade VII Cgi_TALE12  -------------------------PA-----------VVSATIFD---------------------------------------------------------------DAP--------------P-------------
Clade VII Cgi_TALE13  M----------ASRS--------SSPAIAINFTTTLEDIIKATIFD---------------------------------------------------------------DAP--------------P-------------
Clade VII Pfuc_1442   MDI-----------------------SLTITSSTTLNDVIAATIGDLSTTPPDPD---DPTSTNQQSTTPMYSVSYSAPS-----TPQTPTNYSNSPLTQSHSSDFSFDSAPSTPQTPTNYS--NFPLTQIHSSDFSFDS
Clade VII Pfuc_22569  MDI-----------------------SLTITSSTTLKDVIAATIGDLSTTPPDPD---DPTSTNQQSTTPMYSVSYSAPS-----TPQTPTNYSTSPLTQS------------------------------HSSDFSFDS
Clade VII Pfuc_22555  --------------------------PLTITASTTLEDVINATILH---------------------------------------------------------------PQP---------------------------N
Clade VII Pfuc_18402  --------------------------------------------------------------------------------------------------------------------------------------------
Clade VII Pfuc_10095  --------------------------------------------------------------------------------------------------------------------------------------------
Clade VII Pfuc_312    --------------------------------------------------------------------------------------------------------------------------------------------
Clade VII Lgig_162246 MAA---VFPNVSSLPAGNYEVLRFGPQSS---------------------------------------------------------------------------------------------------------------
Clade VII Lgig_162247 MAA---VFPNVSSLPAGNYEVLGFGPQSSED---------CEQVFDTRFEEFYDQEPYSYTGVSIVNLQNLVDE------------RKVVHKDSTHPLETSSIP-------P----------------------------

                            150       160       170       180       190       200       210       220       230       240       250       260       270       280
                    ....|....|....|....|....|....|....|....|....|....|....|....|....|....|....|....|....|....|....|....|....|....|....|....|....|....|....|....|
Clade VI  Pfuc_6013 TLDVIPKCEDISAMLSDVYFDSDD---SYEKPLGAVSLS-DSFTCDSR--FNDSLTSLLDS-PAS--------------TDLP------------------LSGLEHVISDS----SSEFSQDLFNSLV-TQPNV-D---
Clade VI  Cgi_TALE4   SKDVLSRALEFSDLTCLDANSEEQ---SFEEQQPSTEES-HSLTEEPRV-FSDCCFAQADS-DGS---------------SFPPCAVNIMDYEEDLSTCRFSVSLSDLVNEACP-VSAAFCPIIEPSPQ-CSPVT-DSNP
Clade VII Cgi_TALE9   VIATLDSITTEEPPATSSTHSK-----NLTPPQTPPKEF-STSPAEAR--INDALSSLLRS-----------------TTQAP-------------SKKDIFSALQSTLMKECS--QDSTPDVLLQTVY--DPIPHS---
Clade VII Cgi_TALE10  EENDVSIVTNNCQPLSSTPATMRT---QPMAPVTPGNKN-NSFVGDSR--FNDSLSNLLGS-PPP---------------------------ESSLYNNPFFNDLQAALANECTTVKTSLPQSLFDALT-TQPNS-D---
Clade VII Cgi_TALE11  ---------------------------TPEPSTSPVNTSVDTSQAETR--FDECLTSLFSDYPTPEKSHDLHSDLHLALQCLPPTSPKKIFSDSTISSTEIFTALKSIFRSEAD---SSTPSVLIESV---QPEI-S---
Clade VII Cgi_TALE12  ---------------------------TPEPSTSPVNTCVDTSQAETR--FDECLTSLFSDHPTPEKSHDLHSDLHLALQCLPPTSPKKIFSDSTISSTEIFTALKSIFRSEAD---SSTPSVLIKSV---QPEI-S---
Clade VII Cgi_TALE13  ---------------------------SPQLATSPDNTSVDTTQAETR--FDECLNSLFSDHPTPEKSHDLHSDFHLALQCLPLTSPKKIFSDSTISSTEIFTALKSIFRSEAD---SSTPSVLIKSV---QPKI-S---
Clade VII Pfuc_1442   APSTPQTPTNYSNSPVTQFHSSDF---NFDSALSSTPVG---SPPESR--YNDSLYSLHGS-PTS---------------------------NSPFTKDSMLLQLQTKPRDDCT--TYTLP---KSSV---QPLQPS---
Clade VII Pfuc_22569  APSTPQTPTNYSISPVTQFHSSDF---NFDSAVSSTPVG---SPPESR--YNDSLYSLLGS-PTS---------------------------NSPFTKDSMLLQFQTKPREDCT--THTLP---ISSV---QPLPPS---
Clade VII Pfuc_22555  PPTYLPNPREDPTTFTSTHTDSHM---RTPVTTRTQPLTYDTSPAETR--FDDALFSLLDS-PKK---------------------------KTKLTNNPMFRDLQAILKADCS--ADATPNSLFQTLSTANSNN-S---
Clade VII Pfuc_18402  ----------------------------------------------------------------------------------------------------MFRDLQAILKADCS--ADATPNSLFQMLPICKTTN-P---
Clade VII Pfuc_10095  --------------------------------------------------------------------------------------------------------------------------------------------
Clade VII Pfuc_312    --------------------------------------------------------------------------------------------------------------------------------------------
Clade VII Lgig_162246 --------------------------------------------------------------------------------------------------------------EDAT--RTGFPISLFSSV---SNVT-D---
Clade VII Lgig_162247 -------IYEQLXSFIVEDATRTGFPISLFSSVSNVTDV-KNLVDERKVVHKDSTHHLETS-------------------SIP----------------PIYEQLKSFIVEDAT--RTGFPISLFSSV---SNVT-D---

                            290       300       310       320       330       340       350       360       370       380       390       400       410       420
                    ....|....|....|....|....|....|....|....|....|....|....|....|....|....|....|....|....|....|....|....|....|....|....|....|....|....|....|....|
Clade VI  Pfuc_6013 ------IFKRETQLSDRIVKLEPHYPQDIKQLSDFYRYQASLVEMERYKALNQ-GHYSVDHQCKLNLHFDSQLHDIMDRVEQSLCLLEESLRVP----------------------------------------------
Clade VI  Cgi_TALE4   PHINTILFQREQDIRRRFQTLRMDHPQDVQQLSEFYMYQMATIETERLNTTK--PPCLPEHALALNQHYDQQLNQVMDRVEQSIRLLEDARNQS----------------------------------------------
Clade VII Cgi_TALE9   ------VTLRDQRLQTRYNSLQKQYPTEINKLSSFHRHHASIIESDRYMMLHLHAKSSDHFKQMYNAYFDSMLHRMMERVENSLLQLEKTSQVT----------S-----------------------------------
Clade VII Cgi_TALE10  ------VFSREQMLQQRFNSLLPAFPDDIQSLSNFYHHQASEIETERFCAIQQ-KDLPSEVKYSMNFHYDSQLHQIMDRVEQSLMLLEKTEQMS---------FS-----------------------------------
Clade VII Cgi_TALE11  ------PADREQQLLHRFKTLRSFHPQEINDLSSFFKYQSAQVETDRYCALHQNSNQPLHIRESINQHYNITHHRIMDRVESSLQFLEDNLATP----------------------------------------------
Clade VII Cgi_TALE12  ------PADREQQLLHRFKTLRSFHPQEINDLSSFFKYQSAQVETDRYCALHQNSNQPLHIRESINQHYNITHHRIMDRVESSLQFLEDNLATP----------------------------------------------
Clade VII Cgi_TALE13  ------PADREQQLLQRFKTLRSFHPQEINDLSSFFKYQSAQVETDRYCALHQNSNQPLHIRESINQHYNITHHRIMDRVESSLQFLEDNLPTS----------------------------------------------
Clade VII Pfuc_1442   ------PLDRDTLLQQRFATLRTSFPESIEELSTFHRHQSSMLESERRHAL---LNNDAIYHNSLNDYYNYLRHKINDRVERSLAILEVALLID---------SS-----------------------------------
Clade VII Pfuc_22569  ------PLDRDTLLQQRFATLRPSFPESIEELSTFHRHQSSMLESERRHAL---LNNDAIYHNSLNDYCNNLRHKINDRVERSLAILEVALLID---------SS-----------------------------------
Clade VII Pfuc_22555  ------VISRELRLQQRLTKLNAYYPTQITQLSSFHKYQASMVETDRYRELHQIVNHQPTIRTAINHYYEQMLHQIMDRVEISVSTMEEALTTT------------------------------HQPAPTQYSPVINSAP
Clade VII Pfuc_18402  ------VVSRELRLQQRLTKLSDYYPTQVTQLSSFHKYQASMVETDRYRELHQIASHQPTLRNAINHYYDQMLHQIMDRVEMSVGTMETLNTTQ------------------------------YQPAHTQYSPATNSAT
Clade VII Pfuc_10095  -----------------------------------------MVETDRYRELHQIVNHQPSLRTAINHYYDQMLHQIMDRVAISISTMEEALTTTQYQPAPTQNSSVTNSEPTQYSPATNSAPTQYQPTSTQYSPITNSAP
Clade VII Pfuc_312    -----------------------------MEQSTLYIYHAS---------------------------------------------------------------------------------YKFQPMTSQYGQVMSYLD
Clade VII Lgig_162246 ------VKVKEKALLESFDSLRPLYPEPIMQLSCSYKYYSASIEMERLNSIQ--GEL----KDFHNQNFDSQLNKIIDRVEYSLKLLSSRDRPS----------------------------------------------
Clade VII Lgig_162247 ------VKVKEKALLESFDSLRPLYPEPIMQLSCSYKYYSASIEMERLNSIQ--GELSNAMKDFHNQNFDSQLNKIIDRVEYSLKLLSSRDRPS----------------------------------------------
```

```
                               430       440       450       460       470       480       490       500       510       520       530       540       550       560
                        ....|....|....|....|....|....|....|....|....|....|....|....|....|....|....|....|....|....|....|....|....|....|....|....|....|....|....|....|
Clade VI  Pfuc_6013     -------------------------------------------------TKVKTSVR-----------------------------------------------------------SVKPRPLLSKKAVRIMEQWYDDNL
Clade VI  Cgi_TALE4     ------IRP--------------------------------------------------------------------------------------------------------IRPIRKQRASFPKKAILIMEDWYEKNV
Clade VII Cgi_TALE9     ------PKP--------------------------------------------------------------------------------------------------------VKTSLKNRTLLTKKAVDMMEEWYLSNL
Clade VII Cgi_TALE10    ------SRP------------------------------------------------------------------------------------------------------------IKPRPLLSKRAVQLMEKWYDNHL
Clade VII Cgi_TALE11    -------QL----T------------------------------QTVPPQQQTTTQS-----------------------------------------------------------NLRSRPSLSQKAVQLMEEWYYSHR
Clade VII Cgi_TALE12    -------QP----I------------------------------QTVPPPQQTTTQG-----------------------------------------------------------NIRSRPSLSQRAVRLMEEWYYSHR
Clade VII Cgi_TALE13    -------SP----T------------------------------QTASPQQKTTTKS-----------------------------------------------------------NLRSRPSLSQKAVQLMEEWYYSHR
Clade VII Pfuc_1442     ------TKP-----------------------------------ATPRPNQPETPRP-----TQPETPQPTQSETPRPTQPETPTPTRPDTTRRIQPKSLWRTQPET-SETIQQDTPRRARPNLARQAVQLLDDWYRDNM
Clade VII Pfuc_22569    ------TQP-----------------------------------DTPRPTQPETPQP-----TQLETPRHTQRETPEPTQSETPTPTRPDTTRRIQPKSLWRTQPET-SETIQQDTPRRARPNLARKAVQLLDDWYRDNM
Clade VII Pfuc_22555    TQ----YQP----TSTQYPPSTSSAPTQNPLSNNSAPTQYPPATNSAPTQYPSSTNS-----APTQYSPVNNSTPTQYSPVINSAPTQLSPATTSAPT---QYSPVANSSEAPFRLNLRTRPSLSKQAIRLMEVWYHAHI
Clade VII Pfuc_18402    TQ----YSPVTNSAATQYPPFTNLALTQYSPATNSTPTQYSPATNSVPSQYSAATNL-----VPTQYSPATNSAATQYSPATNSVPTQYSPATNSADT---QYSPATFSNEAPFRLNLRTRPSLSKQAIRLMEVWYHAHI
Clade VII Pfuc_10095    TQ----YQP----SSTQYSPVTNSAPTQYPPSTNSAPTQYPPSTNSAPAQYPSTTNS-----APTQYSPVINSASSQYSPDTNSAPTQYSPDTNSAPT---QYSPVTNSSEAPFRLNLRTRPSLSKQAIRLMEVWYHAHI
Clade VII Pfuc_312      TEVYIKYQP----SSSA-PTSTTRPPTQY-----SAPTQYPAATHSASTQYSATTQSQYPVPTPTHYSAPTQTHYSAPTQTQYSVPTQYPMVNYSAPTN-PQTPTQTHSKDAPFKLNLRTRPSLSQRSIRLMEVWYHAHI
Clade VII Lgig_162246   --------P----------------------------------------------------------------------------------------------------------SITRTRPVLTRNSLKVLEEWYECHL
Clade VII Lgig_162247   --------P----------------------------------------------------------------------------------------------------------SITRTRPVLMRNSLKVLEEWYECHL

                               570       580       590       600       610       620       630       640       650       660
                        ....|....|....|....|....|....|....|....|....|....|....|....|....|....|....|....|....|....|....|....|....|
Clade VI  Pfuc_6013     EHPYPTPAAYDAIAVEGGIAVEQVKKWFANKRNRSHNTRSLTEI--AKKKRQIAYATD----QRLNCKLERTLSANCKKEICYSLETGANLFVVTLSIYLVVC--
Clade VI  Cgi_TALE4     SHPYPDSITVELIAIQGGITGEQVKKWFGNKRNRSNNTRTLTEI--AKKKRQIAH----------NCFSDLSL--------------------------------
Clade VII Cgi_TALE9     DHPYPCHKVVQSLAVFGNIREEQVRKWFANKRTRQGRTNKIEKSTPAQ---------------------------------------------------------
Clade VII Cgi_TALE10    EHPYPNIDTIEQLATTGNITPEQVKKWFANKRNRSNNTRTLTEI--AKKKRQLALKN-------MDCYSED----------------------------------
Clade VII Cgi_TALE11    DHPYPPHHIIQDLARKGGVREEQVKKWFSNKRNRSRRSSGVNK------RRSTGV------IKSVPCFW------------------------------------
Clade VII Cgi_TALE12    DHPYPPQHIIQDLARRGGVKEEQVKKWFSNKRNRSRRSSGVNK------RRSTGV------IKSVPCFW------------------------------------
Clade VII Cgi_TALE13    DNPYPPSHIIQDLARKGGVREEQVKKWFSNKRNRSRRSSGVNK------RRSTGV------IKSVPCFW------------------------------------
Clade VII Pfuc_1442     DHPYPGPRTITRLATEGGISEEQVKKWYANKRSRCRNVRPISAI--SQRKRQVRQRKLIFIPVTVLCM-------------------------------------
Clade VII Pfuc_22569    DHPYPGPRTITRLATEGDISEEQVKKWYANKRSRSRNVRPISVI--SQRKRQVRQRKLIFIPVTVLCL-------------------------------------
Clade VII Pfuc_22555    DHPYPNTQDVETLATAGNITEEQVKKWFANKRNRSRNIRPISQI--VRRKRQIQAAAA-NRIIRVPSFC------------------------------------
Clade VII Pfuc_18402    DHPYPDTQDTETLATTGNITEEQVKKWFANKRNRSRNIRPISQI--VRRKRQIQAAA--NRVARVPCLC------------------------------------
Clade VII Pfuc_10095    DHPYPDTQDVETLATAGNITEEQVKKWFANKRNRSRNIRPISQI--VRRKRQIQAATT-NRIIRVPSFC------------------------------------
Clade VII Pfuc_312      DHPYPDTQDVETLATAGNITEEQVKKWFANKRNRSRNIRPMSQI--VRRKRQIQAAATGNRIIRVPSLC------------------------------------
Clade VII Lgig_162246   DHPYPTASQVEWLAQVSSLNTEQVKKWFGNKRSRSKNTRSLTEI--AKVKRRQRLLK----------------------------------------------RH
Clade VII Lgig_162247   DHPYPTASQVEWLAQVSSLNTEQVKKWFGNKRSRSKNTRSLTEI--AKVKRRQRLLK----------------------------------------------RH
```
