# Supplementary material for: Reinforcing the Egg-Timer: Recruitment of Novel Lophotrochozoa Homeobox Genes to Early and Late Development in the Pacific Oyster
Source: Genome Biol Evol. 2015 Jan 27;7(3):677–88. doi: 10.1093/gbe/evv018 (PMC5322547; doi:10.1093/gbe/evv018)
Supplement: Supplementary Data [file supp_evv018_suppl_data.zip › New Microsoft Office Word Document.docx]

# Supplementary Figure legends

**Supplementary Figure 1. Rectangular cladogram displaying the diversity of homeobox genes**. The same tree as Figure 1 (main text), displayed in rectangular format. Due to format limitations, the tree has is split in 2 subtrees: one containing ANTP class representatives (Supplementary Figure 1a) and the other with the rest of homeobox genes (Supplementary Figure 1b). RAxML tree (GTR + Gamma, 1000 bootstraps) with 1940 terminal branches, using the homeobox gene complement of 10 complete bilaterian genomes (eight lophotrochozoans, one ecdysozoan and one deuterostome) and all the homeobox domains annotated for other lophotrochozoans in Pfam (see text). Red branches belong to Pacific oyster sequences.

**Supplementary Figure 2. Gene tree of ANTP class**. RAxML tree (GTR + Gamma, 1000 bootstraps) using only members of the ANTP class. Gene sequences from the rotifer and the leech genome were excluded.

**Supplementary Figure 3. Gene tree of PRD, CUT and PROX classes**. RAxML tree (GTR + Gamma, 1000 bootstraps) using members of PRD, CUT and PROX classes only. Gene sequences from the rotifer and the leech genome were excluded.

**Supplementary Figure 4. Gene tree of TALE, SIX, CERS and HNF classes**. RAxML tree (GTR + Gamma, 1000 bootstraps) using members of TALE, SIX, CERS and HNF classes only. Gene sequences from the rotifer and the leech genome were excluded.

**Supplementary Figure 5. Gene tree of LIM and ZF classes**. RAxML tree (GTR + Gamma, 1000 bootstraps) using members of LIM and ZF classes only. Gene sequences from the rotifer and the leech genome were excluded.

**Supplementary Figure 6. Gene tree of POU class**. RAxML tree (GTR + Gamma, 1000 bootstraps) using POU class homeobox genes only. Gene sequences from the rotifer and the leech genome were excluded.

**Supplementary Figure 7. Alignment of the novel lophotrochozoan-specific homeobox families.** The sequences belonging to new clades (bold font) are aligned with homeobox genes of the same classes from the amphioxus and the beetle genomes.

**Supplementary Figure 8. PADRE domain**. PADRE stands for father in Spanish. The red box demarks the PADRE domain: an 87-amino acid long domain found in the molluscan-specific clades TALE VI and TALE VII.

Supplementary Table 1. **Oyster homeobox genes**. The homeobox gene complement of *Crassostrea gigas* as shown in Table 1, expanded to include class or family defining molecular traits such as sequence motifs or companion domains.

**Supplementary Table 2. Accession numbers of novel homeobox clades in the Lophotrochozoa and molecular synapomorphies.** Expanded version of Table 3 providing accession numbers for representatives of each clade.

**Supplementary File 1. Alignment of 1940 homeobox genes of bettle, amphioxus an 8 lophotrochozoan genomes.** Alignment produced by MAFFT using the E-INS-I algorithm.
