# Supplementary figures and images for: Reinforcing the Egg-Timer: Recruitment of Novel Lophotrochozoa Homeobox Genes to Early and Late Development in the Pacific Oyster
Source: Genome Biol Evol. 2015 Jan 27;7(3):677–88. doi: 10.1093/gbe/evv018 (PMC5322547; doi:10.1093/gbe/evv018)

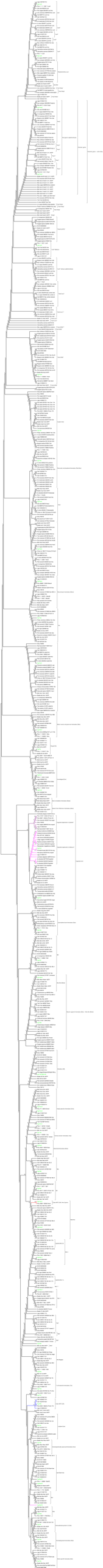

Supplement: Supplementary Data [file supp_evv018_suppl_data.zip › Supp_Figure_2_ANTP.pdf]

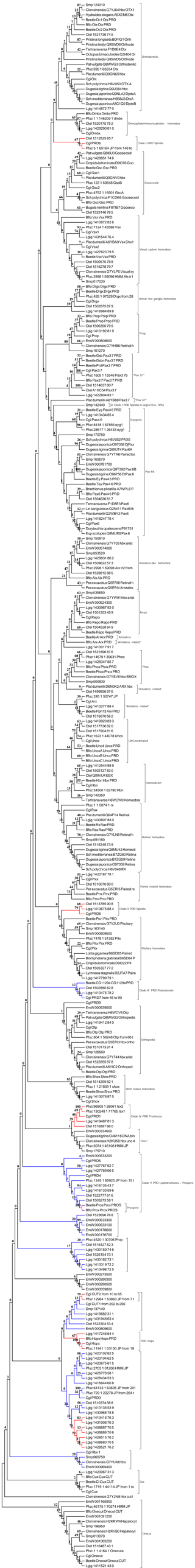

Supplement: Supplementary Data [file supp_evv018_suppl_data.zip › Supp_Figure_3_PRD_OneCUT_Prox_bis.pdf]

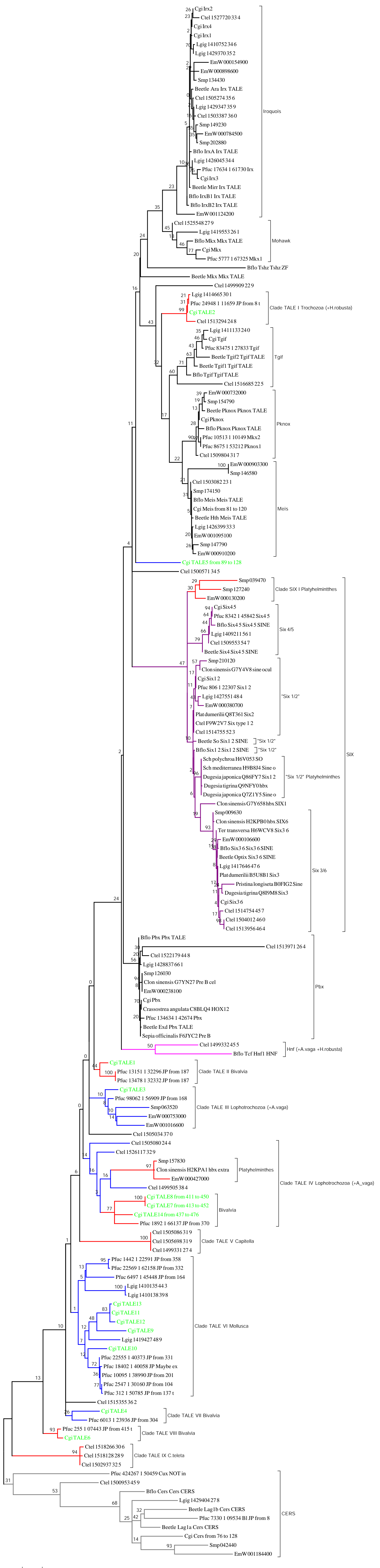

Supplement: Supplementary Data [file supp_evv018_suppl_data.zip › Supp_Figure_4_TALE_SIX_Cers_Hnf.pdf]

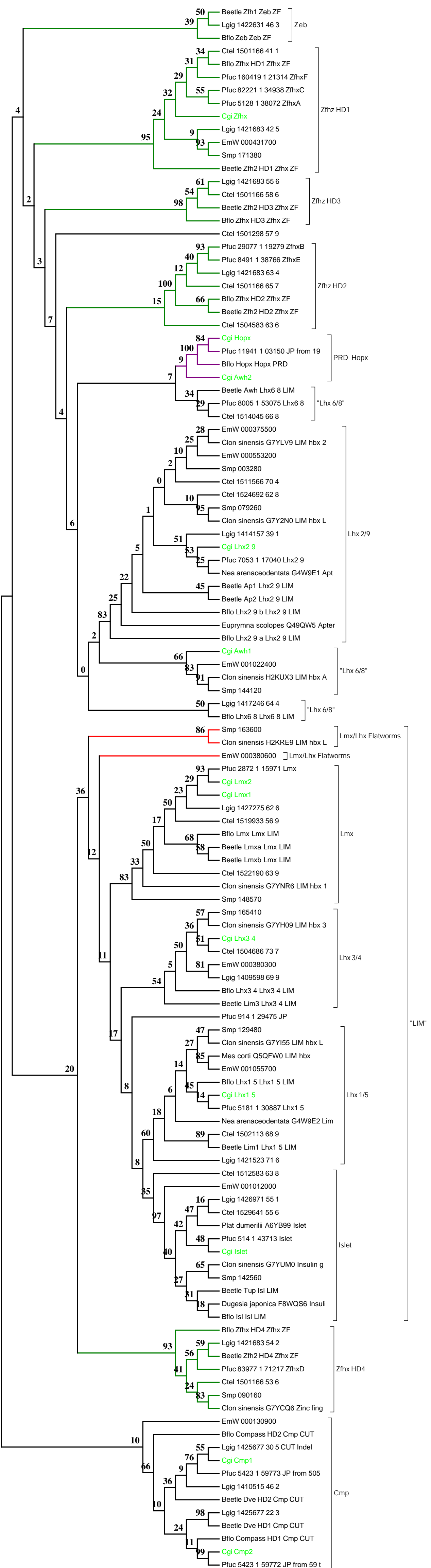

Supplement: Supplementary Data [file supp_evv018_suppl_data.zip › Supp_Figure_5_LIM_ZF.pdf]

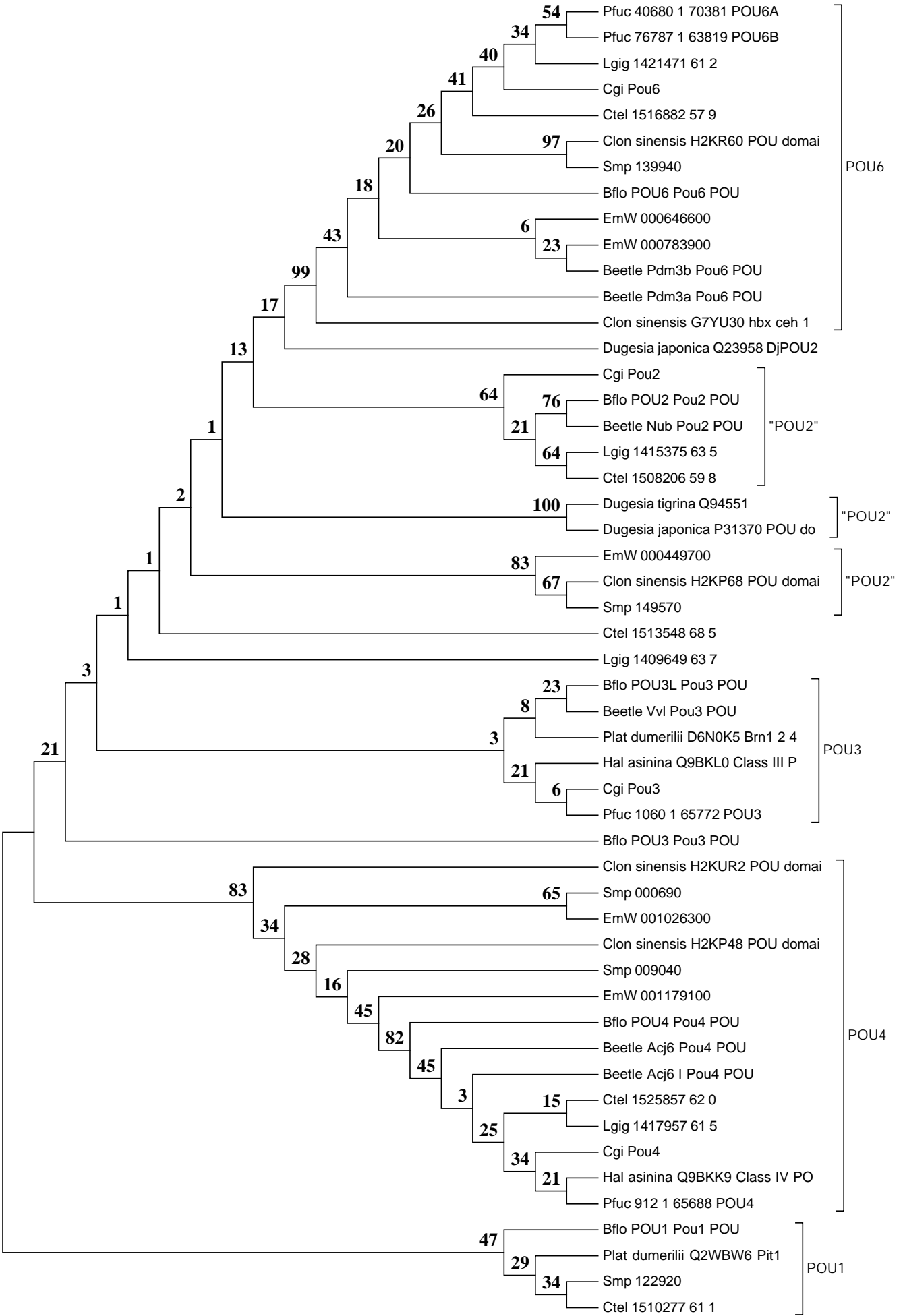

Supplement: Supplementary Data [file supp_evv018_suppl_data.zip › Supp_Figure_6_POU.pdf]
