# Supplementary material for: Reinforcing the Egg-Timer: Recruitment of Novel Lophotrochozoa Homeobox Genes to Early and Late Development in the Pacific Oyster
Source: Genome Biol Evol. 2015 Jan 27;7(3):677–88. doi: 10.1093/gbe/evv018 (PMC5322547; doi:10.1093/gbe/evv018)

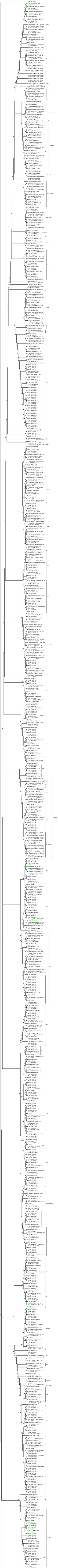

Supplement: Supplementary Data [file Supp_Figure_1a_Largest_tree_ANTP.pdf › Supp_Figure_1a_Largest_tree_ANTP.pdf]
